# Inbreeding does not reduce major histocompatibility complex diversity in the banded mongoose

**DOI:** 10.1186/s12862-025-02456-x

**Published:** 2025-10-14

**Authors:** Nadine Schubert, Hazel J. Nichols, Francis Mwanguhya, Robert Businge, Solomon Kyambulima, Kenneth Mwesige, Michael A. Cant, Jamie C. Winternitz

**Affiliations:** 1 Department of Behavioural Ecology, Bielefeld University, Morgenbreede 45, Bielefeld, 33615 Germany; 2 Department of Biosciences, Swansea University, Singleton Campus, Abertawe, SA2 8PP UK; 3 Department of Evolutionary Population Genetics, Bielefeld University, Bielefeld, Germany; 4 Banded Mongoose Research Project, Queen Elizabeth National Park, Kasese District, Rubirizi, Uganda; 5 Centre for Ecology and Conservation, University of Exeter, Penryn Campus, Penryn, TR10 9FE UK; 6 Evolutionary Immunogenomics, Institute for Animal Cell and Systems Biology, Department of Biology, University of Hamburg, Martin-Luther-King-Platz 3, Hamburg, 20146 Germany; 7 Department of Animal Behaviour, Bielefeld University, Bielefeld, Germany

**Keywords:** MHC, Genetic diversity, Mungos mungo, Balancing selection, Trans-species polymorphism, Carnivore, Phylogenetic mixed model

## Abstract

**Background:**

The major histocompatibility complex’s (MHC) role in the vertebrate adaptive immune response and its exceptional polymorphism make it a key target for studying adaptive gene evolution. However, studies on carnivore MHC often focus on populations with severe bottlenecks or conservation concerns, leading to small sample sizes and unreliable generalizations about MHC diversity. Furthermore, many studies focus on one MHC class and do not cover the whole peptide binding groove of the MHC molecule. Here, we characterize MHC class I (MHC-I) exon 2 and 3, encoding the α1- and α2-domains, as well as MHC-II DRB exon 2 for a large sample (*N* = 285–384) of a wild carnivore of least conservation concern but with high levels of inbreeding, the banded mongoose.

**Results:**

MHC-I showed higher allelic and supertype diversity and polymorphism compared to MHC-II, consistent with findings in humans where MHC-I experiences stronger diversifying selection. MHC-I exon 3 exhibited the lowest diversity, likely due to its specific role in forming the peptide binding groove. Diversifying selection was stronger on MHC-I exon 2 (α1 domain) than exon 3 (α2 domain). Despite frequent inbreeding, banded mongooses showed MHC diversity comparable to other carnivores of least concern using phylogenetic mixed models. Phylogenetic analysis indicated a longer evolutionary trajectory for MHC-II compared to MHC-I and species-specific gene duplication of nonclassical MHC-I sequences clustering with classical sequences. Trans-species polymorphism in nonclassical MHC-I sequences suggested homology or convergent evolution.

**Conclusions:**

This study is the first to characterize both MHC classes of a social, wild carnivore using high-throughput sequencing and a large sample size. Despite frequent inbreeding, banded mongooses exhibit MHC diversity comparable to other carnivores of least conservation concern, challenging assumptions that inbreeding universally reduces genetic diversity. Higher diversity and selection on MHC-I exon 2 emphasize its role in immune defense, while lower diversity in exon 3 highlights functional divergence between the exons. The longer evolutionary trajectory of MHC-II reveals differences in dynamics between MHC classes. Species-specific gene duplication and trans-species polymorphism in nonclassical MHC-I sequences suggest complex evolutionary mechanisms. These findings advance understanding of MHC evolution in wild carnivores, with implications for conservation genetics, particularly regarding the effects of social structure and inbreeding on immune gene diversity.

**Supplementary Information:**

The online version contains supplementary material available at 10.1186/s12862-025-02456-x.

## Background

Understanding how genetic diversity is maintained in natural populations is central to both evolutionary biology and conservation science. Genes involved in immune defense represent key components of this diversity due to their direct role in pathogen recognition and immune response and exhibit an exceptionally high level of polymorphism in both vertebrates and invertebrates [1–3]. In jawed vertebrates the major histocompatibility complex (MHC) plays a crucial role in the adaptive immune response [4]. MHC molecules present intracellular (class I) [5] or extracellular (class II) [6] peptides to T-cells, enabling recognition of infected cells or engulfed pathogens, respectively. Classical MHC molecules, defined by high expression and polymorphism, mediate adaptive immune responses by presenting peptides to T-cells [7, 8]. Nonclassical MHC molecules, while structurally similar to classical ones, have diverse functions including antigen processing, immunomodulation in both innate and adaptive immunity, and even roles unrelated to immune responses [7–9].

A functionally crucial part of classical MHC molecules is the peptide binding region (PBR) which includes the amino acid residues responsible for binding antigens. In both classes the PBR is a dimer built by two domains: MHC-I PBR is formed by α1 and α2 domains (encoded by MHC-I exon 2 and 3) [10] whereas MHC-II is a heterodimer consisting of an α1 and a β1 domain (encoded by MHC-II genes A and B) [11]. We focus on the DR β1 domain because the DR α1 domain is functionally monomorphic in carnivores [12]. Variation in the PBR underpins differential binding of pathogenic peptides, and individuals carrying a wider range of MHC molecules are expected to recognize a broader array of pathogens [13].

Balancing selection maintains MHC diversity through several mechanisms, including heterozygote advantage [14, 15], rare allele advantage [16, 17], and birth-and-death evolution [18]. These mechanisms allow MHC alleles to persist across long evolutionary timescales, often resulting in trans-species polymorphism [19, 20]. Importantly, MHC diversity is often maintained even in small, bottlenecked, or isolated populations that exhibit reduced genome-wide diversity [21–24]. In such contexts, functional measures like MHC supertypes can capture preserved immunological capacity even when allelic richness is low [25, 26]. As such, the MHC represents a crucial axis of adaptive potential, providing a window into the immunogenetic resilience of species of conservation concern.

Carnivores are a particularly valuable group for MHC-focused conservation genomics, as they are disproportionally affected by habitat fragmentation, human conflict, and emerging infectious diseases [27–30]. Many carnivore populations show reduced genome-wide diversity and elevated inbreeding levels [31], raising concerns about their resilience to environmental and pathogenic change. At the genomic level, carnivores typically possess a streamlined MHC architecture, with only one or a few classical MHC class I and II genes [32]. For example, dogs have a single highly polymorphic MHC-I gene (DLA-88; [33]), while domestic cats and giant pandas each have three (cats: FLAI-E, -H, and -K [34]; giant pandas: Aime-C, -I, and -L [35]). Consistent with this low gene copy number, many carnivore species exhibit modest MHC allelic diversity. However, many studies focus on bottlenecked or threatened populations, such as wild dogs [36], Ethiopian wolves [37], and red wolves [38]), where small sample sizes may limit generalizations about MHC diversity. For example, the critically endangered Siberian (Amur) tiger is monomorphic at the MHC-II DRB locus [39], while other tiger subspecies similarly exhibit low MHC allelic richness, a trend common among endangered felids [40].

Conversely, widespread carnivore species with large populations, such as North American raccoons, can exhibit high MHC variation, with 66 unique MHC-II DRB alleles identified among 246 individuals [41]. Pinniped carnivores such as harbor seals and gray seals also maintain elevated MHC diversity, attributed to expanded gene families and multiple MHC class I loci [42]. These contrasting patterns underscore the need to include data from non-threatened but inbred species in comparative analyses, to better disentangle the respective impacts of population history, inbreeding, and sampling effort on observed MHC variation. In this context, examining MHC diversity can offer insights into retained immunogenetic variation that may buffer populations against disease and environmental change [21, 23]. Comparative immunogenomics across carnivores further enables the identification of general evolutionary patterns and lineage-specific adaptations in MHC architecture and diversity.

The banded mongoose (*Mungos mungo*) offers a powerful model for addressing these questions. It is a cooperatively breeding carnivore that combines high reproductive output with frequent close-kin mating. Social groups contain 10–40 adults, many of which reproduce synchronously, producing large communal litters raised by the group [43–45]. High natal philopatry, weak kin recognition, and limited kin avoidance contribute to high levels of inbreeding, with 66.4% of individuals showing evidence of inbreeding and 7.1% resulting from matings between first-degree relatives [46]. Inbreeding depression has been documented [46, 47], and while banded mongooses show signs of kin-biased mate avoidance, mechanisms of mate choice remain unclear [48–50]. In addition to its social and genetic structure, the banded mongoose is increasingly relevant from a conservation standpoint due to emerging disease threats, including outbreaks of *Mycobacterium mungi* and *M. bovis* [51–54], highlighting the importance of understanding immune gene variation in this species.

Despite being listed as a species of least concern, the banded mongoose thus provides a unique opportunity to investigate MHC evolution in a system where cooperative social structure, regular inbreeding, and pathogen pressures intersect. Here, we use a targeted next-generation sequencing approach to characterize diversity and selection at classical MHC loci in the banded mongoose. Specifically, we (i) sequence MHC-I exons 2 and 3 and MHC-II DRB exon 2 to examine polymorphism in the peptide binding regions, (ii) test for signatures of natural selection, (iii) assess the presence of trans-species polymorphism, and (iv) compare MHC diversity in banded mongooses to other carnivores using phylogenetically informed analyses.

## Methods

### Study site and population sampling

Data were collected between 1994 and 2018 from a wild population of banded mongooses inhabiting Queen Elizabeth National Park in Uganda (0°12’S, 27°54E’). The study area covered approximately 10 km² savannah including the Mweya peninsula and the surrounding area. The population contained approximately 10–12 social groups, corresponding to approximately 250 individuals alive at one point. Banded mongooses were captured in Tomahawk traps (Tomahawk Live Trap Co., Tomahawk, WI) and were anesthetized with isoflurane as described in detail Jordan et al. [55]. A 2-mm tissue sample for genetic analysis was taken from the tip of the tail using sterile surgical scissors. A diluted solution of potassium permanganate was then applied to the wound to minimize risk of infection. Tissue samples were stored in 96% alcohol until further processing. All animals were given a tattoo or a subcutaneous pit tag (TAG-P-122IJ, Wyre Micro Design Ltd., UK) to allow permanent identification. Animals were then allowed to recover in a covered trap with access to water. Following this, they were released together with other members of their social group at the site of capture.

### Ethical statement

Research was conducted under approval of the Uganda National Council for Science and Technology, and all procedures were approved by the Uganda Wildlife Authority and the Ethical Review Committee of the University of Exeter. All research procedures adhered to the ASAB Guidelines for the Treatment of Animals in Behavioral Research and Teaching [56].

### Genetic analyses

#### DNA extraction

We extracted DNA from 465 individuals using Qiagen^®^ DNeasy blood and tissue kits (Quiagen, the Netherlands) according to the manufacturers protocol. DNA was stored in the provided elution buffer or TE buffer and kept frozen at −20 °C until further processing.

#### Amplification of MHC genes with high-throughput sequencing

The extracted DNA was used to amplify fragments of MHC-I exon 2, MHC-I exon 3, and MHC-II DRB exon 2 of banded mongooses. Primers for MHC-I exon 2 were established based on the felid sequences published by Yukhi & O’Brien [57], whereas the primers of MHC-I exon 3 and MHC-II DRB exon 2 were newly designed based on the published sequences of other closely related carnivore species (NCBI accession numbers in Table S1). Briefly, carnivore sequences were aligned with MAFFT v7 [58], and primers were designed using Primer3 [59]. Candidate primer pairs were tested on dam-sire-offspring triads; resulting PCR products were cloned (TOPO TA, Invitrogen) and Sanger sequenced (mean 14 clones/product, range 4–26) to confirm offspring alleles matched parental genotypes. Final primer pairs were chosen based on amplification success and product length (Table S2). These primers amplified the 271-bp fragment (228-bp excluding primers) of MHC-I exon 2 (forward: CCACTCCCTGAGGTATTTCTACACC, reverse: CTCACCGGCCTCGCTCTG), the 270-bp fragment (232-bp excluding primers) of MHC-I exon 3 (forward: GGTCACACAGCATCCAGAGA, reverse: GCTGCAGCGTCTCCTTCC), and the 239-bp fragment (201-bp excluding primers) of MHC-II DRB exon 2 (forward: CGAGTGCCATTTCACCAACG, reverse: GCTGCACCGTGAAGCTCT). We added 10 bp tag sequences with a minimum edit distance of 7 to our primers [60], allowing each sample to be uniquely identified. This enabled us to pool all PCR products from a plate and use a single Illumina adapter per pool, while still keeping track of individual samples during de-multiplexing and processing. To improve sequencing quality, we also added 6–8 bp of random ‘junk’ sequence at the 5’ end of each primer to reduce issues caused by identical bases being sequenced in a row. Each complete primer had the structure: 5’-junk–tag–primer–3’.

We amplified every sample in duplicate and ran a negative control in a differently positioned well within each plate. PCR amplification methods and NGS library construction are described in detail in the Supplementary Materials. Normalized NGS libraries were run on an Illumina MiSeq^®^ System (Illumina Inc, United States) with a MiSeq Reagent Kit v2 (500 cycles) according to the manufacturer’s protocol at the Max Planck Institute for Evolutionary Biology, Plön, Germany. The run on the Illumina MiSeq^®^ System included 30 libraries stemming from our experiment and 6 belonging to another experiment. Index sequences that were used for multiple libraries could be uniquely assigned to libraries, as the libraries differed in primer pairs used. To test for contamination in our workflow, we assigned negative controls to each plate and had at least one negative control per library. These controls confirmed that none of our libraries showed signs of contamination.

#### Clean-up and processing of MHC

We de-multiplexed the raw data obtained from the illumina MiSeq runs using the AmpliSAS/AmpliCHECK genotyping tools developed by Sebastian et al. [61]. We first merged paired-end reads using AmpliMERGE and then removed low quality and erroneous sequences with AmpliCLEAN with a Phred quality score of 30. Demultiplexing and filtering with AmpliCLEAN resulted in 2,036,358 reads. We then used AmpliCHECK to explore the data and identify which advanced program parameters, such as minimum allele frequency and maximum number of alleles per amplicon, should be modified in the AmpliSAS step. Minimum allele frequency was estimated by comparing the frequency of artefacts with the frequency of putative alleles. We then tested different parameter settings and performed plausibility checks using preexisting pedigree data [47, 50] that gave us information on the genotypes of parent-offspring triads. For each exon we selected the median allele frequency of artefacts identified in AmpliCHECK as the minimum frequency threshold. We then determined the reproducibility between samples and their duplicates using the formula [(Number of shared alleles*2)/sum of alleles in replicates] to identify the best settings for AMPLISAS (Table S3). After running AmpliSAS, we used AmpliCOMPARE to combine the results for technical replicates.

We then performed further manual quality and plausibility checks with the sequencing data obtained from the AmpliSAS pipeline described above. We first estimated the difference in bases between all sequences. We then used this data to follow the step-by-step procedure depicted in Figure S1. The number of base pairs that differed, the depth at which the sequence occurred, the number of samples the sequence appeared in, whether it appeared only together with a different, highly similar sequence, as well as whether it was present in both replicates of a sample were considered to classify a sequence variant either as a putative allele or an artefact. Sequences that were present in only one of the duplicate samples were kept for that individual if they fulfilled the criteria set for a putative allele in the step-by-step allele validation procedure described above.

Before manual allele validation, 70 sequences were retained for MHC-I exon 2, 16 sequences for MHC-I exon 3, and 23 sequences for MHC-II DRB exon 2. After clean-up following step-by-step the procedure described in the previous paragraph, we were left with 37 sequences for MHC-I exon 2 (32 coding, 5 pseudogenes), 14 sequences for MHC-I exon 3 (all sequences are coding, but three of these have the same amino acid sequences as other sequences in the set, leaving 11 different amino acid sequences), and 17 for MHC-II DRB exon 2 (15 coding sequences, 2 pseudogenes).

We further used Megablast [62] on NCBI GenBank to investigate similarity of the putative alleles to known carnivore sequences to validate the sequences. Next, we translated putative alleles, checked for stop codons and investigated whether conserved sites known to have structural importance can be found in the sequences [63, 64]. We then used the results of the sample duplicates to calculate reproducibility of the genotypes.

We tried to assign alleles to loci using MHC typer V1.1 using a maximum-likelihood approach for reconstructing haplotypes while considering null alleles or copy number variation (CNV), identical alleles shared between loci as well as deviations from Hardy-Weinberg-Equilibrium [65], but were unsuccessful. Detailed methods for the approach used can be found in the Supplementary Material.

#### Comparison of carnivore MHC diversity

Banded mongooses inbreed frequently, which may affect their levels of genetic diversity. To compare the total number of MHC sequences found in banded mongooses with the MHC population diversity of other carnivore species, we searched for studies reporting the total number of MHC sequences for MHC class I exon 2 and exon 3 as well as MHC class II DRB. We used the search engines Google Scholar and PubMed and the combined search terms “MHC”, “diversity” and “carnivore” (search performed: August 22nd 2024). In addition, we searched for sequence similarity of our sequences in GenBank using Megablast [62] and looked for linked literature for the carnivores that matched our MHC sequences. We identified 78 observations of 39 species for which we compiled metadata on sample size, MHC class, number of exons amplified, and conservation status (Supplementary Data 1). We assigned a conservation status (least concern (LC), near threatened (NT), vulnerable (VU), endangered (EN) or critically endangered (CR)) using the IUCN Red List of Threatened Species version 2024−1 [66] as a proxy for threat of extinction.

### Statistical analyses

#### Sequence polymorphism

We assessed sequence polymorphism through the number of polymorphic sites, average number of nucleotide differences, total number of mutations, nucleotide diversity, and pairwise identity using DnaSP 6.12.03 [67]. We estimated the amino acid p-distance with uniform rates in MEGA-X [68]. A detailed description of the methods can be found in Winternitz et al. [69].

#### Recombination

RDP4 v.4.101 software [70], which uses several distinct algorithms aimed at detecting recombinant sequences, was used to infer recombination signals in MHC-I exon 2 and 3 and MHC-II DRB exon 2. We used the following methods to infer recombination in our data set: RDP [71], GENECONV [72], BootScan [73], SiScan [74], Maxchi [75], Chimaera [76], and 3Seq [77]. First, we screened the alignments of MHC-I and MHC-II nucleotide sequences for recombination with an automated exploratory search using the default settings, a statistical significance threshold of *p* = 0.05, and Bonferroni correction for multiple comparisons. We retained a recombination event for further analysis when it was detected by two or more algorithms. Second, we continued with a manual examination based on the guidelines of Martin et al. [78]. Therefore we sequentially examined all detected recombination events based on following criteria: the recombination event could be detected in more than one sequence, the characteristics of the recombination event (e.g. the identity of the recombinants and the breakpoint positions) could be verified, an average p-value across the recombinant sequences below 0.05 for the method detecting recombination, and no warning that the putative recombination signal has been caused by evolutionary processes other than recombination. A recombination event was accepted when all these criteria were fulfilled and rejected if this was not the case.

#### Inference of selection

Selection was determined based on comparing rates of nonsynonymous (dN) and synonymous (dS) substitutions. Positive (diversifying) selection is characterized by (dN > dS) driving changes in the amino acid sequence, whereas (dN < dS) represents negative (purifying) selection, and (dN = dS) implies neutral evolution.

We identified codon sites under natural selection by determining rates of dN and dS for each site based on two complimentary methods: FUBAR and MEME. FUBAR (Fast, Unconstrained Bayesian AppRoximation) infers rates based on a Bayesian approach [79]. It detects evidence of pervasive selection by assuming constant pressure. However, individual sites can experience different levels of positive and negative selection (episodic selection) and methods that can only detect pervasive selection will miss these effects. MEME (Mixed Effects Model of Evolution) uses a mixed-effects maximum likelihood approach instead to identify individual sites with signs of pervasive and episodic selection [80]. Positively selected sites (PSS) were thus those detected by either FUBAR (pervasive selection) or MEME (pervasive and episodic selection). Those detected by FUBAR only were classified as negatively selected sites. For analysis with FUBAR, posterior probability >0.9 was set as our significance threshold, as these values strongly indicate natural selection. For analysis with MEME, our significance threshold was set to < 0.05. Both FUBAR and MEME selection inference was carried out on the Datamonkey server [81], https://www.datamonkey.org/, last accessed: January 18, 2024.

To benchmark these findings against a widely used method and provide standardized reporting, we also conducted a complementary analysis with EasyCodeML [82], a wrapper for PAML’s CODEML [83]. This approach requires a coding sequence alignment and a corresponding phylogenetic tree. We aligned classical MHC coding sequences for each exon using MAFFT v7.490 [58, 84]. Phylogenetic trees were generated with IQ-TREE via its webserver ([85] http://iqtree.cibiv.univie.ac.at), using ModelFinder [86] to select the best-fit model by BIC (MHC-I exon 2: HKY + F + G4; MHC-I exon 3: F81 + F + I; MHC-II: F81 + F + G4). Trees were constructed with 5000 ultrafast bootstrap replicates [87, 88]. In EasyCodeML, we ran codon site models using the nested model comparison mode, specifically testing M7 vs. M8 and M8a vs. M8. M7 assumes only purifying or neutral selection, while M8 allows a proportion of sites to evolve under positive selection (dN/dS >1). M8a constrains this class to neutrality (dN/dS = 1). We used likelihood ratio tests (LRTs) to evaluate model fit and applied Bayes Empirical Bayes (BEB) to identify positively selected sites (* = posterior ≥ 0.95, ** ≥ 0.99).

We estimated mean dN-dS rates with standard error averaging over all sequence pairs using 1000 bootstrap replicates as a measure of strength of selection. For all classes and exons, analyses were carried out based on the Nei-Gojobori model with Jukes-Cantor correction for multiple substitutions [89]. All analyses determining the strength of selection as rates of (dN-dS) were performed in MEGA X [68].To compare positions of the identified positively selected codon sites to those of the human PBR, the obtained amino acid sequences for MHC-I exon 2, exon 3, and MHC-II DRB exon 2 were aligned per exon and visually compared to PBRs identified in crystallographic analyses of human MHC molecules [10, 11]. Since recombination can lead to overestimating positively selected sites, recombination inference was performed using RDP v.4.101 [70] before determining selection patterns.

#### Supertype classification

Supertypes were estimated by clustering unique, functional amino acid sequences based on functional similarity. For each exon, the Sandberg distance [90], which uses five physio-chemical z-descriptor values, was calculated at each positively selected site (PSS) using the R package *Peptides* [91] to generate a similarity matrix. We then applied find.clusters() with criterion = “goodfit” and method = “kmeans”. This procedure was repeated 500 times, and the mean, mode, and median cluster numbers were used to define clusters for each exon.

Alleles were assigned to groups using discriminant analysis of principal components (DAPC; dapc() in the *adegenet* R package [92]. This process was also repeated 500 times to estimate repeatability using Light’s Kappa from the *irr* package [93] and the mean assignment proportion for each exon. For comparison, we also assigned functional alleles to supertypes using human-identified peptide binding residues (PBRs) [10, 11], following the same clustering procedure described above.

#### Linkage disequilibrium analysis

We conducted linkage disequilibrium (LD) analysis to aid in identifying haplotype groupings, estimating the number of loci, and characterizing genetic structure and diversity within the population. Strong positive correlations between alleles likely indicate that they belong to different loci on the same haplotype, forming a haplotype block. In contrast, strong negative correlations suggest that alleles may come from the same locus or from different, mutually exclusive haplotypes. Additionally, the recurrence of the same allele combinations across individuals points to a limited number of common haplotypes in the population. LD was assessed using the Phi coefficient, calculated as the Pearson correlation for binary data, implemented via the R package corrplot [94]. To visualize correlations and linkage groups we used the *corrplot* function with the options: order = ‘hclust’ and hclust.method = ‘ward.D’.

#### Phylogenetic relationships

To investigate the phylogenetic relationships among MHC sequences of banded mongoose and closely related carnivore species, we searched reference sequence genes (RefSeqGenes) available on NCBI GenBank (search performed on July 10, 2024). Sequences in this RefSeqGene set are intended to be well-supported, exist in nature, and represent a prevalent, ‘normal’ allele. The sequence search used the key terms (“histocompatibility antigen“[All Fields] AND (“class I“[All Fields] OR “class II“[All Fields])) AND “carnivores“[porgn] AND “srcdb refseq“[Properties] AND alive[prop]). We selected MHC-I and MHC-II DRB genes from 12 carnivore species, five feliformia (cat-like) and seven caniformia (dog-like). These included the following families: Felidae (cats), Herpestidae (mongooses), and Hyaenidae (hyenas); Canidae (dogs), Ursidae (bears), Mustelidae (weasels, badgers, otters, and related species), Otariidae (sea lions and fur seals), and Phocidae (true seals); Feliforms: cheetah (*Acinonyx jubatus*), domestic cat (*Felis catus*), Canada lynx (*Lynx canadensis*), striped hyena (*Hyaena hyaena*), and meerkat (*Suricata suricatta*); Caniforms: dog (*Canis lupus*), giant panda (*Ailuropoda melanoleuca*), American black bear (*Ursus americanus*), Eurasian badger (*Meles meles*), domestic ferret (*Mustela putorius*), California sea lion (*Zalophus californianus*) and gray seal (*Halichoerus grypus*)). Human MHC-I genes and human and carnivore MHC-II DQB genes were selected to serve as outgroups to root trees. Since coding regions are more evolutionarily conserved than introns, we extracted the coding sequence (CDS) from each RefSeq gene for alignment and tree construction. We also included sequences of characterized MHC genes from the NCBI nucleotide database for *Felis catus* [95, 96] and *Acinonyx jubatus* [97]. Sequences of known nonclassical MHC genes (FLAI-A, FLAI-M, FLAI-O, FLAI-Q, FLAI-J, FLAI-L [95, 96]; DLA-12, DLA-64 [98]; AIME-1906 [99], HLA-E, HLA-F, HLA-G [100]) were included in the alignments to see if they clustered with banded mongoose sequences with strong support, suggesting homology and thus that we may have amplified nonclassical banded mongoose MHC sequences.

We aligned 107 sequences with 2109 nucleotide sites for MHC-I (Supplementary Data 2) and 61 sequences with 831 nucleotide sites for MHC-II (Supplementary Data 3) using MAFFT v7.490 [58, 84]. Maximum likelihood phylogenetic trees for MHC sequences were created using the IQ-TREE webserver ([85] http://iqtree.cibiv.univie.ac.at). We first used ModelFinder [86] to select the top supported model based on BIC (MHC-I best-fit model = TVM + F + R4; MHC-II best-fit model = GTR + F + R3), and then used IQ-TREE with 5000 ultrafast bootstrap (UFBoot) alignments to build the trees [87, 88]. To compare the inferred MHC gene trees with the species tree, 100 trees based on the Mammal birth-death node-dated completed tree [101] were downloaded from https://vertlife.org/ for the 14 species in this study. Consensus topology and average branch lengths were computed with the consensus.edges function from the R package phytools v2.3-0 [102] using 50% majority rule consensus. Phylogenetic tree figures were created using the R package ggtree v3.12.0 [103]. Analyses and figures produced in R used version 4.4.1 [104].

#### Phylogenetic regression of carnivore MHC diversity

To compare the total number of MHC sequences found in banded mongooses with the MHC population diversity of other carnivore species, we conducted a Bayesian phylogenetic linear mixed model with the R package brms [105]. We used a phylogenetic consensus tree estimated using the R package phytools [106] of 100 phylogenetic trees (https://vertlife.org/phylosubsets/) dated by tip with mean branch lengths [101]. Species not listed in the tree were assigned to the nearest relative to compute a phylogenetic covariance matrix including all species. To predict how total number of alleles (log_10_ transformed) was related to extinction risk, we included the fixed effects of conservation status (ordinal), log_10_ transformed sample size, the number of exons sequenced (1 or 2), MHC class (I or II), the random effects of species to account for multiple observations per species and a covariance matrix of the relationship between species from branch lengths to account for non-independence among species. Our model used a gaussian distribution, default priors, 2 chains, 6000 iterations, 1000 warmup iterations, and the parameter adapt_delta increased to 0.99 to eliminate the number of divergent transitions during sampling. Model predictions were obtained using the R package ggeffects [107] and plotted using the R packages ggeffects, gginnards [108], ggrepel [109], ggnewscale [110], and ggplot2 [111].

## Results

### Genotyping results

All sequences that were verified as either classical or nonclassical putative alleles can be found in the supplementary material.

#### MHC-I exon 2

**Fig. 1:**
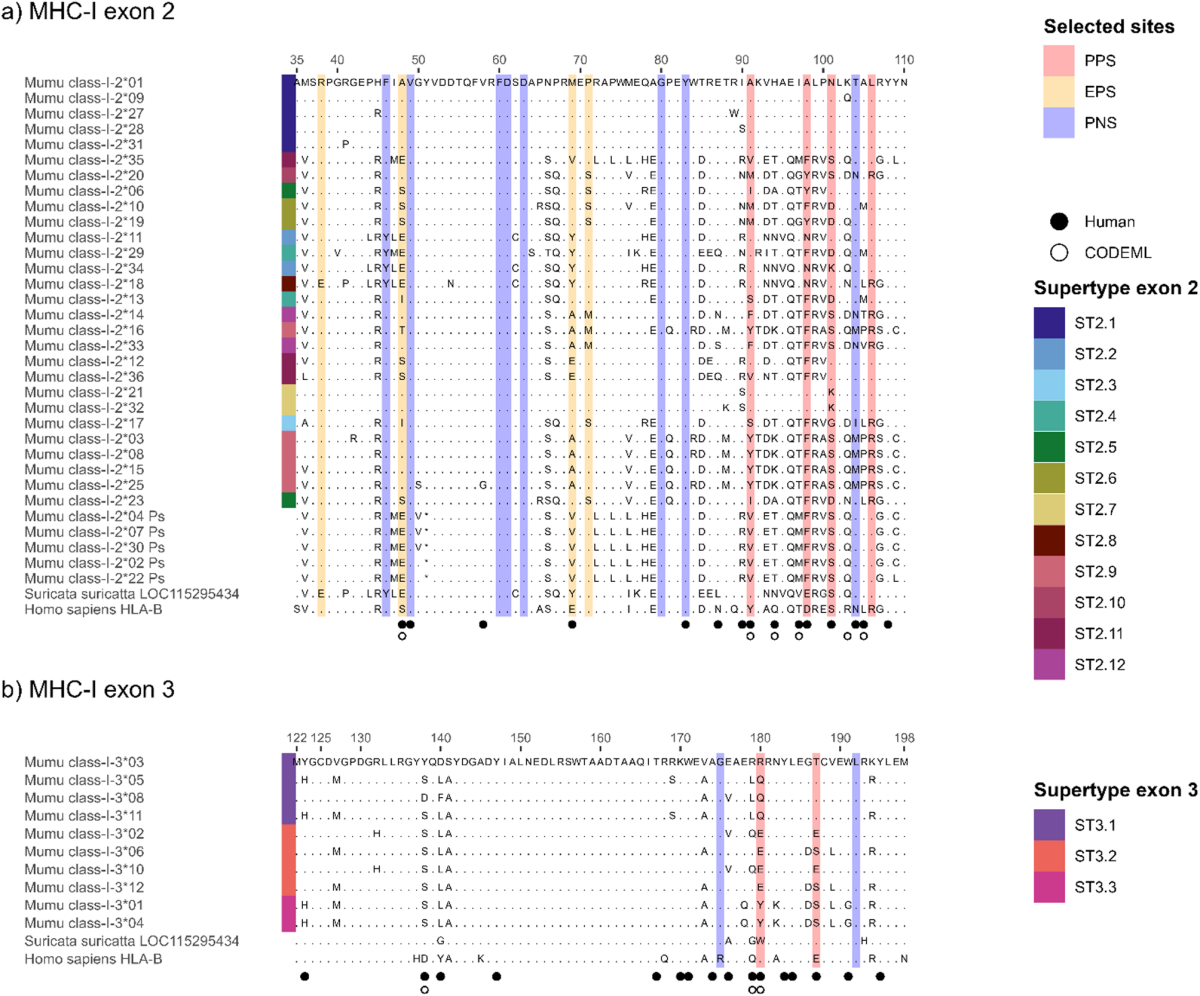
Alignment of amino acid sequences for classical MHC class I alleles of the banded mongoose (*Mungos mungo).* Displayed are the alignments for MHC-I exon 2 (**a**) aligned with HLA-B (accession number NC_000006, chromosome 6 assembly accession number NC_000006.12) and the meerkat (*Suricata suricatta*) MHC class I histocompatibility antigen sequence (accession number LOC115295434, genome NC_043706). Residues are numbered from 35 to 110 based on the HLA-alignment. MHC-I exon 3 amino acid sequences have been aligned with the same human and meerkat sequences as MHC-I exon 2, but the residue numbering established based on the HLA-sequence reaches from 122 to 198 (**b**). Banded mongoose sequences are available on GenBank with the accession numbers PQ137681 - PQ137731. Dots represent sites that are identical to the amino acid shown at this site in the sequence on the top of the alignment. Filled circles indicate human peptide binding residues based on Saper et al. [10]. Open circles indicate CODEML positive selection sites. PPS: pervasive positive selection, EPS: episodic positive selection, PNS: pervasive negative selection. Supertypes are indicated on the left as colored bars. Pseudogenes were not included in the selection or supertype analysis but are shown for visualization purposes

For MHC-I exon 2, 37 putative alleles of 228 bp length were detected (Fig. 1a). Apart from allele 17, all alleles had BLAST hits (E < 6e^−65^) with feliformia MHC-I exon 2 loci between 86.4% and 99.56% identity (please see supplementary files for detailed blast results). Allele 17 also had BLAST hits (E < 6e^−65^) but only with mammal species other than feliform species, with the highest sequence identity of 94.26% and E = 2^−90^ for the horse *Equus caballus*. Apart from five putative alleles (alleles 02, 04, 07, 22 and 30) all sequences translated without stop codons (on reading frame 1) and showed high conservation of sites known to be structurally important in other species’ classical MHC-I loci (Table S4). To classify the putative alleles as classical or nonclassical, we used the human MHC sequence (accession number AAA76608.2) as a reference, aligned it with our putative alleles and compared the nucleotides of the known conserved sites [63] with those from our putative alleles. Site Y84 from Kaufman et al. [63] corresponded to site 108 in the HLA and banded mongoose alignment and was conserved in all putative alleles. In contrast site 83, which corresponded to HLA Y59, was not conserved in allele 05, 24, 26 and 37. These four alleles were thus classified as putatively nonclassical MHC sequences so were not considered for further selection analyses. Unfortunately, gene expression data was not available for this study, although showing patterns of restricted expression would strengthen our characterization of nonclassical alleles.

#### MHC-I exon 3

For MHC-I exon 3, 14 putative alleles of 232 bp length were detected (Fig. 1b). All these alleles had BLAST hits (E < 1e^−5^) with feliform MHC-I exon 3 loci between 92.58% and 99.57% (please see supplementary files for detailed blast results). The alleles translated without stop codons (on reading frame 1) and showed high conservation of sites known to be structurally important in other species’ classic MHC-I loci [10]. Allele 01 and 04, allele 06 and 12, and allele 07 and 13 only contained synonymous substitutions and translated into the same amino acid sequence, respectively. Similar to MHC-I exon 2, we investigated whether putative alleles of exon 3 had conserved sites that were known from human MHC [63]. HLA-A residue T143 from Kaufman et al. [63] corresponded to site 167 of the HLA/banded mongoose alignment and was conserved in all putative alleles (Table S4). In contrast Y171, which corresponded to site 195 in the mongoose sequence, was not conserved in allele 07, 09, 13 and 14. These four alleles were thus classified as nonclassical MHC sequences so were not considered for further selection analyses.

#### MHC-II DRB exon 2

**Fig. 2:**
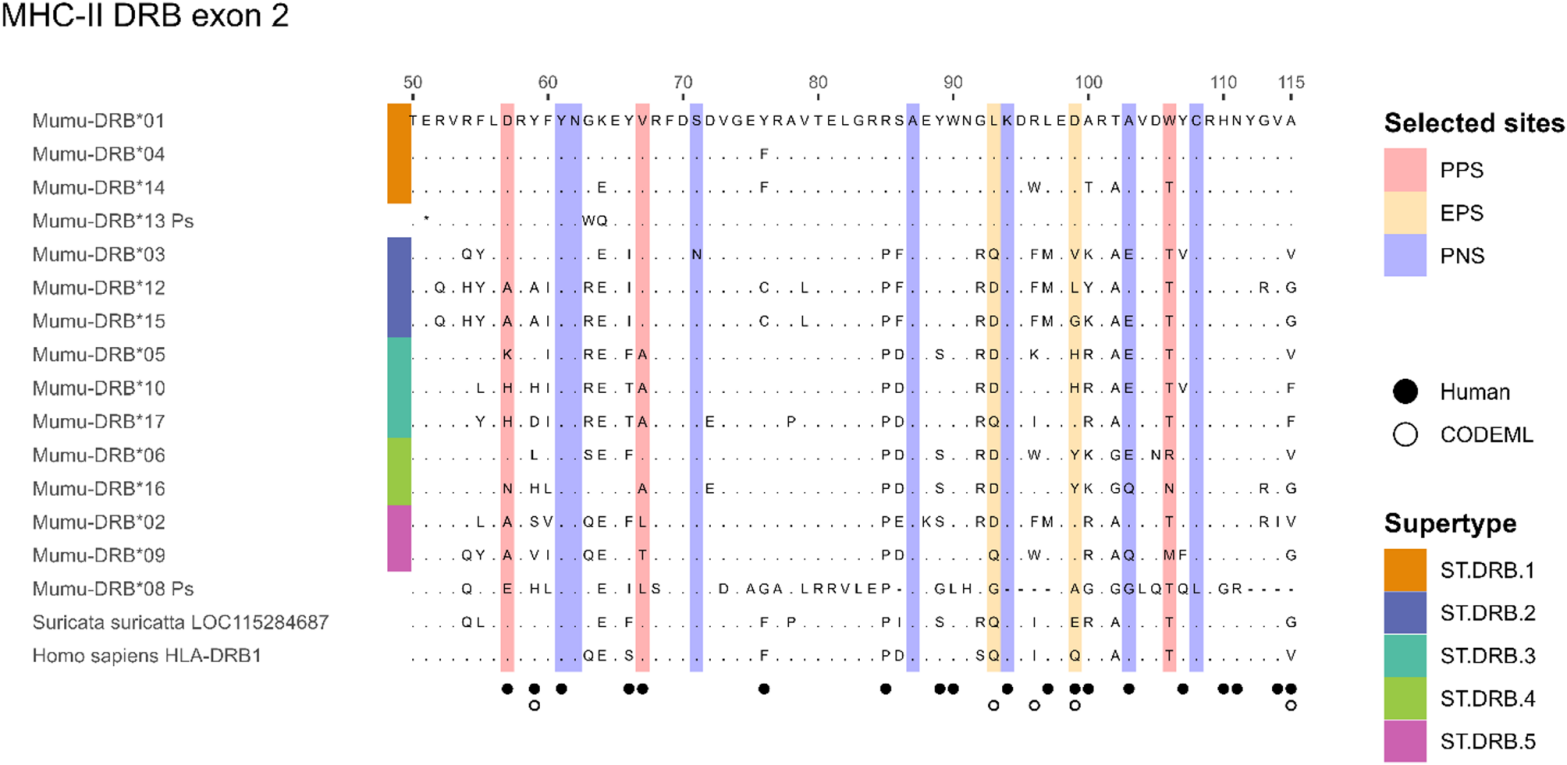
Alignment of amino acid sequences for classical MHC class II DRB exon 2 alleles of the banded mongoose (*Mungos mungo).* Alignments were made with HLA-DRB1 (genome accession number NC_000006) and DLA class II histocompatibility antigen DR-1 beta chain-like of the meerkat (*Suricata suricatta*) (accession number LOC115284687, genome accession number NC_043706). Residues are numbered from 50 to 115 based on the HLA-alignment. Banded mongoose sequences are available on GenBank with the accession numbers PQ137732 - PQ137748. Dots represent sites that are identical to the amino acid shown at this site in the sequence on the top of the alignment. Filled circles indicate human peptide binding residues based on Brown et al. [11]. Open circles indicate CODEML positive selection sites. PPS: pervasive positive selection, EPS: episodic positive selection, PNS: pervasive negative selection. Supertypes are indicated on the left as colored bars. Pseudogenes were not included in the selection or supertype analysis but are shown for visualization purposes

For MHC-II DRB exon 02, 17 putative alleles of 201 bp length were detected (Fig. 2). Apart from allele 05 and 16 all alleles had BLAST hits (E < 1e^−5^) with feliform MHC-II loci between 86.53% and 95.52% identity (please see supplementary files for detailed blast results). Allele 05 was most similar to the sequence of the bamboo lemur *Hapalemur griseus griseus* (E = 1^−71^, % ident. = 92.86) and allele 16 had the best match with a sequence from the Himalayan bear *Ursus thibetanus* (E = 2^−64^, %ident. = 91.1). Except for allele 08 and 13, all alleles translated without stop codons (on reading frame 3). The sequences showed high conservation of sites known to be structurally important in MHC-II sequences of other species’ classical MHC-II loci [11, 112]. We investigated whether putative alleles of DRB exon 2 had conserved sites that were known from human MHC [63]. Conserved site H81 from Kaufman et al. [63] corresponded to site H110 in the HLA sequence retrieved from GenBank (accession number NP002115.2) and the aligned mongoose sequence and was conserved in all putative alleles (Table S4). In contrast W61, which corresponded to site 90 in the HLA/mongoose sequence alignment, was not conserved in allele 07 and 11. These two alleles were thus classified as nonclassical MHC sequences that were not considered for further selection analyses.

### Assigning alleles to loci

We tried to assign alleles to loci for MHC class I and II but were unable to establish the same assignment with the same optimal BIC over multiple runs. We also acknowledge that henceforth we use the term ‘allele’ to designate sequence variants from unknown loci. MHC nomenclature from this study takes the first 2 characters of the genus species name (Mumu) following the recommendations of Maccari et al. [113]. For class I, the four-letter species abbreviation is followed by class-I and the respective exon, either − 2 or −3. The last part of the sequence nomenclature for both classes is the sequence number, ordered by decreasing population frequency. Finally, the abbreviation Ps (pseudogene) indicates non-coding sequences.

### Supertype classification

Assigning functional alleles to supertypes using banded mongoose positively selected sites (PSS) resulted in 12 clusters for MHC-I exon 2, 3 for MHC-I exon 3, and 5 for MHC-II DRB exon 2 (Figs. 1 and 2). Repeatability was perfect (Kappa = 1) across all exons. The mean assignment proportion based on PSS was 0.96 for MHC-I exon 2, and 1.0 for both MHC-I exon 3 and MHC-II DRB exon 2.

Assigning functional alleles to supertypes using human-identified PBRs yielded 11, 4, and 5 clusters for MHC-I exon 2, exon 3, and MHC-II DRB exon 2, respectively, each with perfect repeatability and mean assignment proportions of 0.89, 1, and 0.92. As mean assignment proportions were consistently higher using PSS defined from the banded mongoose, we present results based only on PSS-derived supertypes.

### Individual allelic and supertype diversity

For MHCI-I exon 2, 321 samples were successfully genotyped with a mean reproducibility of 89.4%. Read number per sample ranged from 100 to 5000 reads, with a mean of 637 reads per sample. Allele numbers per individual ranged from 3 to 14 alleles with a median of 9. For MHC-I exon 3, 285 individuals were successfully genotyped with a mean reproducibility of 96.6%. Read number per sample ranged from 110 to 5000 reads, with a mean of 1215 reads. Allele numbers per individual ranged from 1 to 5 with a median of 1. For MHC-II DRB exon 2, 384 individuals were successfully genotyped with a mean reproducibility of 97.7%. Read numbers varied from 101 to 5000 reads per sample with a mean of 1224 reads. Individuals had between 1 and 7 alleles, with a median of 3.

Supertype numbers based on PSS ranged from 2 to 8 (median = 5) for the MHC-I exon 2, from 1 to 3 (median = 1) for the MHC-I exon 3, and from 1 to 5 (median = 2) for the MHC-II DRB exon 2. Number of unique supertypes inferred using mongoose PSS and human PBR were strongly correlated (MHC-I exon 2: df = 319, p-value < 2.2e-16, cor = 0.94; MHC-I exon 3: df = 280, p-value < 2.2e-16, cor = 0.96; MHC-II DRB exon 2: df = 383, p-value < 2.2e-16, cor = 0.79), suggesting the two methods predict consistent numbers of functional groups per individual.

Numbers of within-individual alleles and supertypes were also strongly correlated (MHC-I exon 2: cor = 0.779; MHC-I exon 3: cor = 0.788; MHC-II DRB exon 2: cor = 0.825; all p-value < 2.2e-16), indicating that high allelic diversity in the banded mongoose equates with high functional (supertype) diversity.

**Fig. 3:**
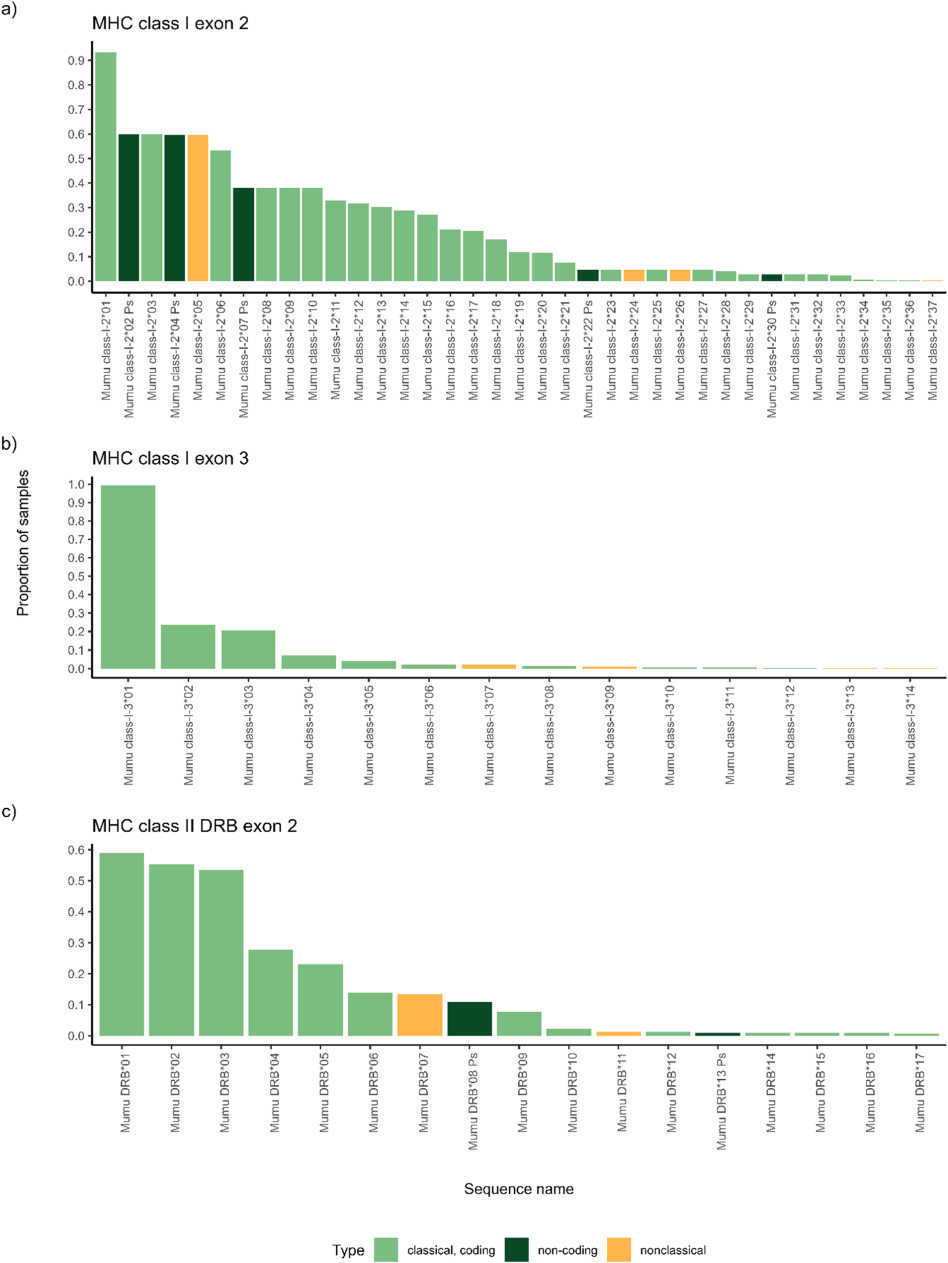
Allele frequencies for MHC-I and -II. The frequency with which the identified putative alleles appear in individuals is displayed. Alleles are either identified as nonclassical (orange) or classical sequences (green) for MHC-I exon 2 (**a**), for MHC-I exon 3 (**b**) and for MHC-II DRB exon 2 (**c**). Within the classical alleles, non-coding sequences are highlighted (dark green)

The frequencies of the putative alleles strongly differ between the genes investigated (Fig. 3). Whereas MHC-II DRB exon 2 genotypes are dominated by coding sequences (Fig. 3c), MHC-I exon 2 genotypes include non-coding sequences at a much higher rate (Fig. 3a). The two pseudogenes Mumu class-I-2*02-Ps and − 04-Ps appear in more than half of the individuals in this study. In contrast, Mumu-DRB*08-Ps appears in a tenth of the tested individuals and Mumu-DRB*13-Ps only in 1% of the samples. For MHC-I exon 3 no non-coding sequences were identified (Fig. 3b).

This variation in allele frequencies is also reflected at the functional level, with marked differences in supertype distribution across loci. For example, MHC-I exon 2 included supertype ST2.1, found in all individuals, alongside ST2.7, which occurred in only 8% of individuals. MHC-I exon 3 showed a similarly skewed distribution, with ST3.3 present in 99% of individuals and ST3.1 in only 23%. In contrast, MHC-II DRB exon 2 displayed a more balanced distribution of supertypes (14%–78%, Fig. S2).

### Sequence polymorphism

MHC-I exon 3 had the highest number of nucleotides with 232, followed by MHC-I exon 2 with 228 nucleotides and MHC-II DRB exon 2 with 201 nucleotides. For MHC-I exon 2, 28 different alleles were found with 100 polymorphic sites and 132 mutations in total (Table 1). The average number of nucleotide differences was k = 32.238 and the nucleotide diversity Pi = 0.1414. The mean amino acid p-distance was 0.251 ± 0.029. For MHC-I exon 3, diversity was overall lower with 10 distinct alleles, 30 polymorphic sites and 36 mutations detected. The nucleotide diversity for MHC-I exon 3 was Pi = 0.0565 and the mean amino acid p-distance equaled 0.111 ± 0.025. For MHC-II DRB exon 2, 13 distinct alleles were detected with 79 polymorphic sites and 105 mutations. The nucleotide diversity was Pi = 0.1544 and the mean amino acid p-distance was 0.259 ± 0.037. Pairwise identity of MHC-I exon 2 was 85.3% and for exon 3 94.2%, which corresponds to a divergence of 14.8% and 5.8% respectively. For MHC-II DRB exon 2 pairwise identity was 84.3% resulting in a divergence of 15.7%. Consequently, both MHC classes and all exons fulfill the minimum requirement of 5% sequence divergence and thus the power required for the detection of recombination by most methods.

**Table 1:** Sequence polymorphism of banded mongoose MHC-I and MHC-II. S: number of polymorphic sites, eta: total number of mutations, k: average number of nucleotide differences, pi: nucleotide diversity, AA p-distance: number of amino acid differences per site

MHC class	Exon	Nucleotide number	Allele number	S	Eta	K	Pi	AA *p*-distance
MHC-I	2	228	28	100	132	32.238	0.1414	0.251 ± 0.029
MHC-I	3	232	10	30	36	13.111	0.0565	0.111 ± 0.025
MHC-II	DRB 2	201	13	79	105	31.026	0.1544	0.259 ± 0.037

### Recombination

For both MHC classes and all exons no recombination event met our validation threshold. This means that according to our criteria no sequence is considered a recombination product of other sequences since no breakpoints were confirmed by the algorithms used by GARD or RDP4.

### Inference of selection

MHC-I exon 2 showed stronger signs of selection compared to MHC-I exon 3 and MHC-II DRB exon 2 (Table 2). FUBAR and MEME detect pervasive and episodic selection respectively and found 8 sites under positive selection for MHC-I exon 2 compared to 2 in MHC-I exon 3 and 5 in MHC-II DRB exon 2. Similarly, model M8 in CODEML was significantly better than competing models that did not allow positive selection (Table S5), and it found 6, 3, and 5 sites under positive selection for MHC-I exon 2, exon 3, and MHC-II DRB exon 2, with 2/6, 1/3, and 2/5 matching PSS detected by FUBAR and MEME. Of CODEML’s sites, 5/6, 2/3, and 3/5 matched human PBR sites for MHC-I exon 2, exon 3, and MHC-II DRB exon 2, respectively (Figs. 1 and 2, Table S6 & S7).

A similar pattern among exons can be observed for negative selection, which was detected with FUBAR for 8 sites in MHC-I exon 2, for 7 in MHC-II DRB exon 2 and for 2 sites in MHC-I exon 3. Negatively selected sites mostly showed no polymorphism at all and were fixed for a single amino acid, e.g. at site 60, 61 and 63 for MHC-I exon 2, at site 192 for MHC-I exon 3 and at site 61 and 62 for MHC-II DRB exon 2. However, there were also polymorphic sites under negative selection, e.g. site 103 of MHC-II DRB exon 2 that we also identified to represent a PBR site in the HLA-sequence. Interestingly MHC-I exon 3 was fixed for a G (glycine, hydrophobic) in banded mongooses as well as meerkats but the HLA-B sequence showed an R (arginine, basic) at this site.

The strength of selection defined as the ratio of non-synonymous vs. synonymous mutations did not show a clear pattern (Table 2), with no significant selection found when considering all sites, nor for sites corresponding to the human PBR. However, when considering the positively selected sites (PSS) only, significant positive selection could be detected for both classes and all exons.

**Table 2:** Patterns of selection detected in MHC class I exon 2 and 3 and MHC class II DRB exon 2 of banded mongooses. dN-dS: ratio between non-synonymous and synonymous describing the direction of selection, PSS: positively selected site, PBR: peptide binding region. Significant p-values are in bold

MHC class	Exon	*n*_alleles_ (*n*_AAsites_)	Number of sites under selection		All sites			PSS				Human PBR			
			Positive	Negative	dN - dS		*P*	dN - dS	*P*			dN - dS		*P*	
MHC-I	2	28 (76)	8	8	0.908	0.183		5.554	8*10 ^−8^			0.917	0.180		
MHC-I	3	10 (77)	2	2	1.129	0.131		2.402	**0.009**			0.164	0.051		
MHC-II	DRB 2	13 (66)	5	7	−0.042	1		5.146	1.6*10 ^−7^			0.281	0.390		

### Linkage disequilibrium analysis

**Fig. 4:**
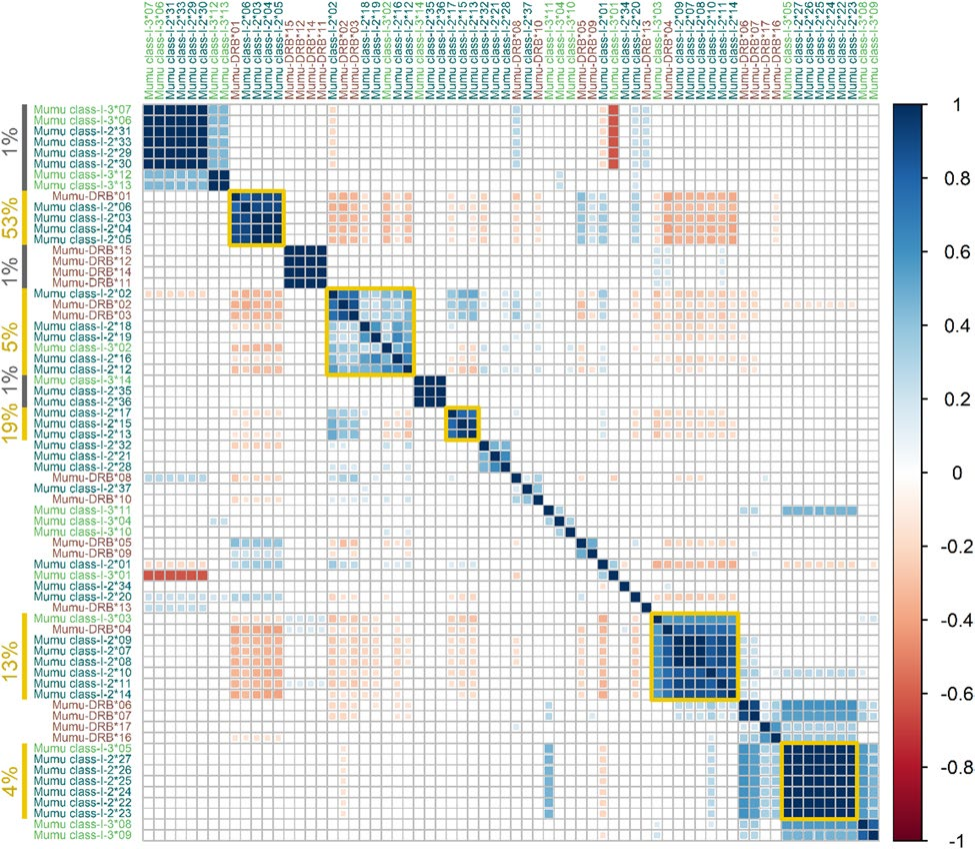
Linkage disequilibrium among MHC alleles reveals haplotype structure. Pairwise correlations between alleles from MHC-I exon 2 (dark green labels), MHC-I exon 3 (light green labels), and MHC-II DRB exon 2 (maroon labels) are shown. Only significant correlations (*p* < 0.05) are displayed, with color indicating strength and direction: strong positive correlations appear as dark blue squares, and strong negative correlations as deep red squares. Allele clusters with strong negative correlations to other clusters, suggesting they belong to alternative haplotypes, are highlighted with gold borders. Colored vertical bars indicate the alleles comprising each cluster. Percentage of samples with specific allele combinations appear next to vertical bars indicating the allele cluster. The number of distinct alleles per exon within each cluster suggests the presence of at least six loci for MHC-I exons 2 and 3, and at least four loci for MHC-II DRB

We performed linkage disequilibrium (LD) analysis to identify haplotype groupings, estimate locus number, and characterize genetic structure in the population. Strong positive correlations among up to six MHC-I exon 2 alleles and up to four MHC-II DRB exon 2 alleles suggest at least six and four loci per haplotype, respectively. Conversely, strong negative correlations between high frequency allele clusters indicate mutually exclusive haplotypes. Notably, specific allele combinations occurred in 1% up to 53% of samples, indicating a limited number of common, high-frequency haplotypes in the population (Fig. 4). These LD-defined clusters also corresponded to the most frequent individual alleles observed (Fig. 3), reinforcing the role of haplotype structure in shaping allele frequency distributions.

### Phylogenetic analysis

**Fig. 5:**
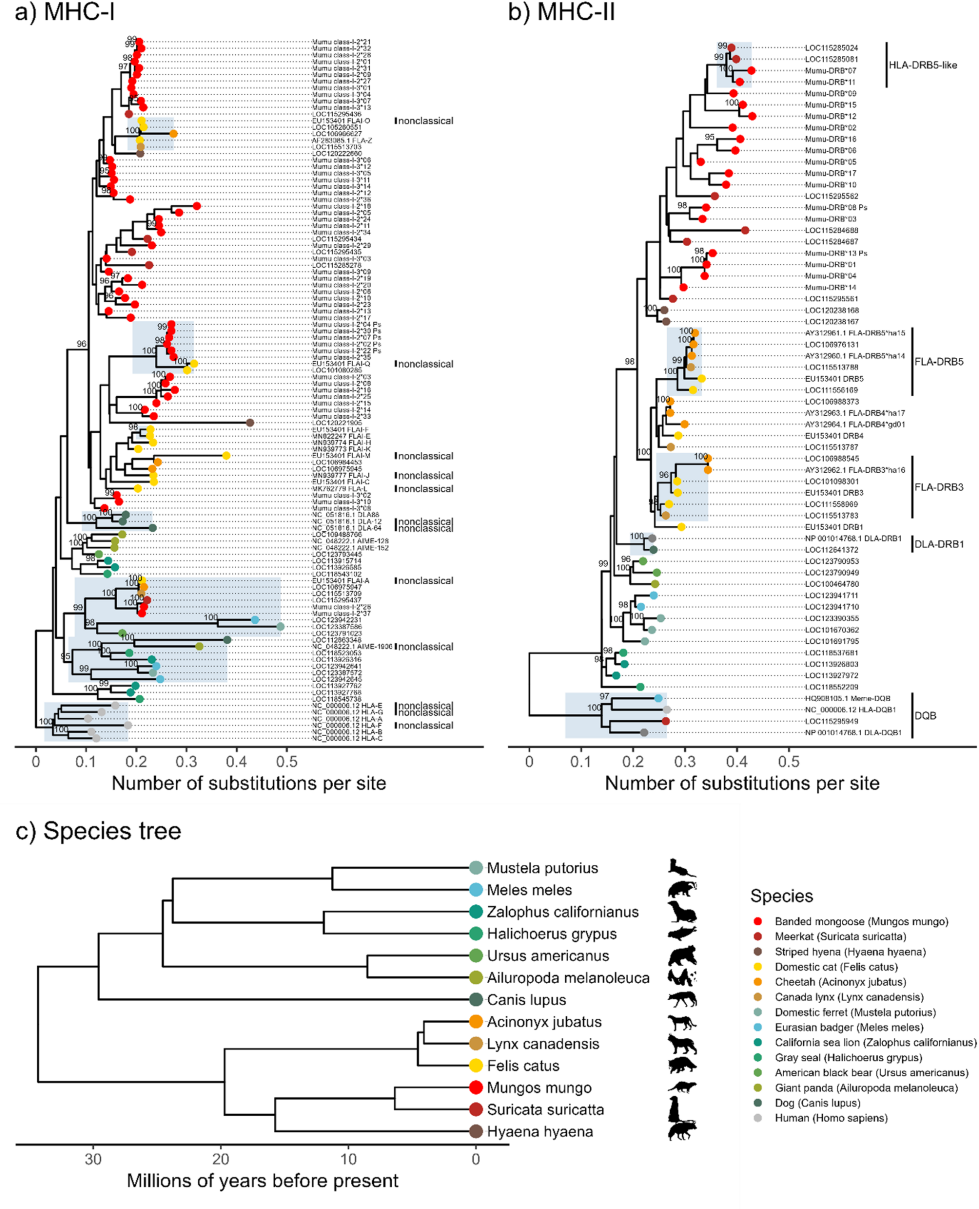
Phylogenetic relationships of banded mongoose MHC-I and MHC-II sequences across carnivores. Maximum-likelihood phylogenetic trees were created for banded mongoose MHC sequences using RefSeq CDS of MHC alleles from diverse carnivore species. UFBoot support values greater than 95% are shown―the recommended cutoff for high probability that a clade is true. **a** A phylogenetic tree including 37 and 14 *Mungos mungo* sequences of MHC class I exon 2 and 3, respectively. The tree was rooted using the human HLA class I allele clade as an outgroup. Clades with strong support that include known nonclassical loci are highlighted. Banded mongoose sequences cluster both interspecifically and by species or with the closest relative, the meerkat. **b** A phylogenetic tree including 17 *Mungos mungo* sequences of MHC class II DRB exon 2, rooted by a clade of MHC-II DQB sequences. Known locus-specific clades with strong support are highlighted, illustrating that MHC-II loci tend to have deeper evolutionary history than MHC-I loci, with duplicated MHC-II loci shared across species. **c** The phylogenetic consensus tree of 13 species included in the MHC sequence trees. Tips are color-coded so that feliformia (cat-like carnivores) have warm colors (red-yellow) and caniformia (dog-like carnivores) have cool colors (blue-green). Artwork from phylopic.org

To investigate the phylogenetic relationships among MHC sequences of banded mongooses and related carnivore species, we created MHC gene sequence trees and compared these with the species tree. We found that sequences mostly clustered with high (≥ 95%) probability within species and within families (Felidae (cats), Herpestidae (mongooses), and Hyaenidae (hyenas); Canidae (dogs), Ursidae (bears), Mustelidae (weasels, badgers, otters, and related species), Otariidae (sea lions and fur seals), and Phocidae (true seals)) (Fig. 5). The suborders feliformia (cat-like species) tended to cluster independently, with exceptions for MHC-I including a clade with a known nonclassical cat locus (FLAI-A) and predicted nonclassical loci in the Eurasian badger and domestic ferret (Patr-E-like). The exceptions to clustering by suborder feliformia for MHC-II were the DQB and DRB locus clades. Therefore, within our trees there are some homologous MHC-I loci shared among carnivores but most evolved after divergence at the family level. MHC-II loci appear to have longer evolutionary history than MHC-I, with the DQB clade including sequences from a primate and carnivores, the DRB clade spanning carnivores, and orthologous loci clustering by family.

Banded mongoose MHC-I sequences mostly clustered by species or with meerkats, but one well-supported clade included noncoding sequences and the cat nonclassical locus FLAI-Q, and another included putative nonclassical sequences Mumu class I-N*26 and 37 and the cat nonclassical locus FLAI-A (Fig. 3a). Clades with strong support that include known nonclassical loci (FLAI-O, FLAI-Q, FLAI-A, AIME-1906) are highlighted to show that these include multiple species and could suggest homology or convergent evolution. Other known nonclassical loci (FLAI-F, DLA-12, DLA-64, HLA-E, HLA-F, HLA-G) cluster with classical loci within species, suggesting species-specific gene duplication and subsequent divergence. Thus, banded mongooses may have both homologous (interspecific) and orthologous (species-specific) nonclassical loci, as predicted by the conserved residue motifs described in Sect. 3.1.1–3.1.3.

For MHC-II, banded mongoose sequences form a monophyletic clade with meerkats and striped hyena, suggesting MHC DRB loci from these species were present in a common ancestor approximately 17.7 million years ago [101]. Meerkats appear to have six DRB loci based on incomplete genomic scaffolds (NCBI RefSeq assembly: GCF_006229205.1). Banded mongooses have at least four DRB loci based on a maximum of seven NGS sequences recorded per individual, though we were not able to use phylogenetic inference to identify individual loci from our 202–205 bp sequences.

### Comparative analysis of carnivore MHC diversity

To investigate whether our sample size was sufficiently large to capture the majority of the allelic diversity of our study population, we conducted an accumulation analysis using the package *vegan* version 2.6–8.6. The accumulation curves (Fig. S3a-c) showed that the samples sizes used in this study were sufficient to capture the vast majority of allelic diversity. We thus do not think that the sample size led to underestimation of allelic diversity.

**Fig. 6:**
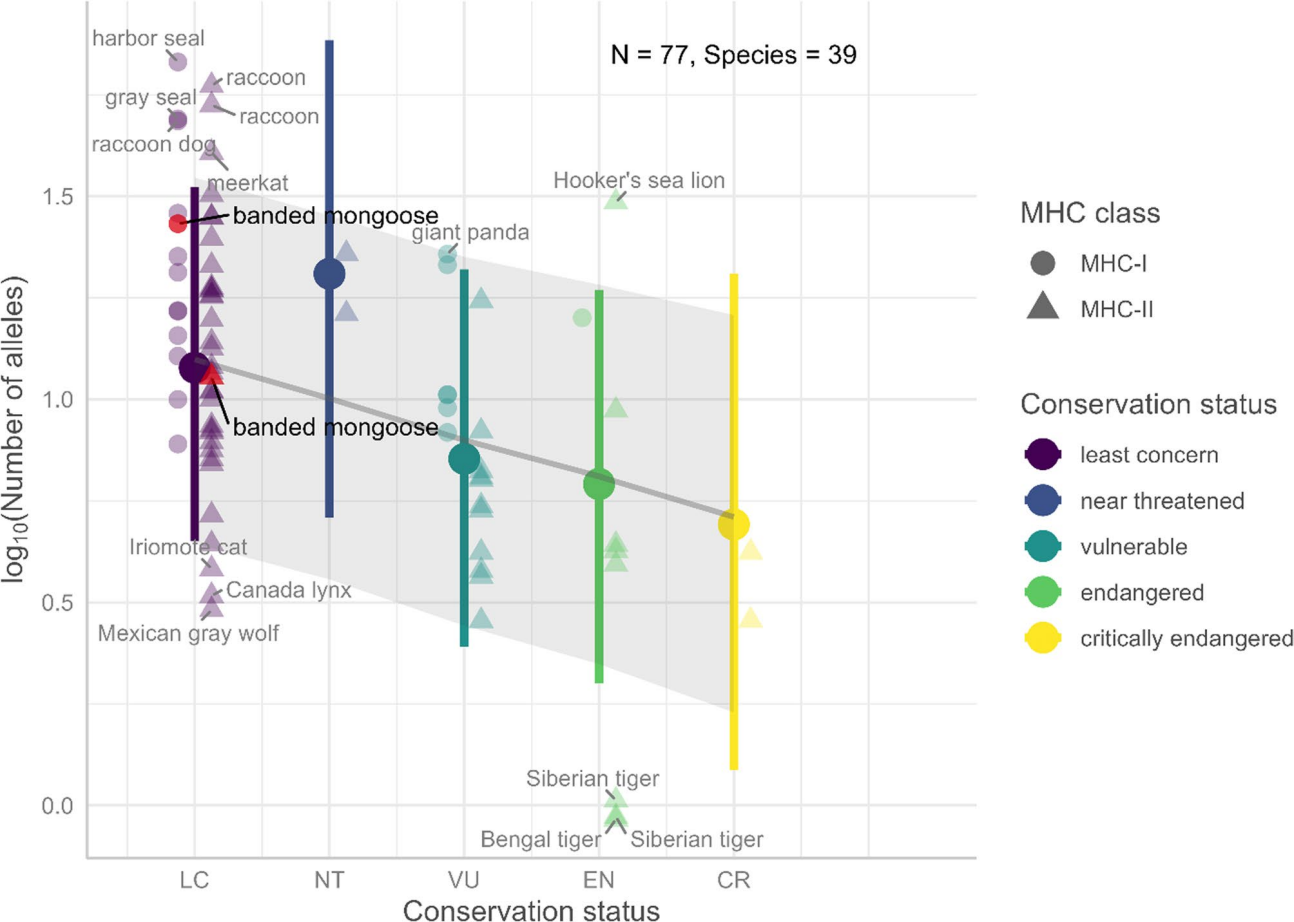
Predicted MHC allele number decreases with increasing conservation threat. Large colored points with 95% credible intervals (CI) show predictions from a Bayesian phylogenetic linear mixed model, controlling for species, phylogeny, MHC class, and sampling effort (number of individuals and exons). The linear trend and 95% credible interval of log₁₀ allele number by numeric conservation status is shown for interpretation. Raw data are displayed separately for MHC-I and MHC-II. Banded mongoose values, highlighted in red, fall within the 95% CI for species of least concern. Species with values outside the predicted 5–95% CI range per conservation status are labeled

To compare banded mongoose population MHC allelic diversity with those of other carnivore species, we needed to control for sampling effort, phylogenetic relationships, and population size. We compiled a complete dataset of 77 observations of 39 carnivore species with MHC sequence data (consensus phylogenetic tree with conservation status and relative allelic diversity as Fig. S4). The phylogenetic signal representing the proportion of variation in a trait attributed to phylogenetic effects was very low and the credible interval included zero (λ = 0.02, 95% CI [0 ‒ 0.08]), indicating that closely related species do not have more similar MHC allelic diversity than expected by chance, suggesting limited influence of evolutionary history on MHC diversity. Controlling for species and phylogenetic relatedness, we found as expected that the number of MHC alleles genotyped increased with sampling effort (posterior probabilities = 0.26 [0.16 ‒ 0.37]), that there were fewer alleles genotyped for MHC-II than MHC-I (−0.36 [−0.50 ‒ −0.20]), and that the number of alleles showed a significant linear decreasing trend as threat of extinction increased (−0.41 [−0.74 ‒ −0.09], Fig. 6).

We found no evidence that banded mongooses have reduced MHC diversity at either class after accounting for sampling effort and conservation status. Their total allele counts (MHC-I: 28 alleles, 75th percentile; MHC-II: 13 alleles, 54th percentile) fall well within the expected range for species classified as “least concern.” In contrast, several “least concern” species were outliers at the lower end, including the historically bottlenecked Canada lynx (*Lynx canadensis*), the island-endemic Iriomote cat (*Prionailurus bengalensis iriomotensis*), and the Mexican gray wolf subspecies (*Canis lupus baileyi*). Among “endangered” species, extremely low MHC-II diversity was observed in both the Siberian (*Panthera tigris altaica*) and Bengal tigers (*Panthera tigris tigris*), each with just one allele identified from very limited samples. At the higher end of MHC diversity among “least concern” species, the raccoon (*Procyon lotor*) and meerkat (*Suricata suricatta*) showed elevated MHC-II allele counts, while the harbor seal (*Phoca vitulina*), gray seal (*Halichoerus grypus*), and raccoon dog (*Nyctereutes procyonoides*) exhibited relatively high MHC-I diversity. Similarly, the “vulnerable” giant panda (*Ailuropoda melanoleuca*) and the “endangered” Hooker’s sea lion (*Phocarctos hookeri*) displayed relatively high MHC-I and MHC-II diversity for their threat categories, respectively.

Large colored points with 95% credible intervals (CI) show predictions from a Bayesian phylogenetic linear mixed model, controlling for species, phylogeny, MHC class, and sampling effort (number of individuals and exons). The linear trend and 95% credible interval of log₁₀ allele number by numeric conservation status is shown for interpretation. Raw data are displayed separately for MHC-I and MHC-II. Banded mongoose values, highlighted in red, fall within the 95% CI for species of least concern. Species with values outside the predicted 5–95% CI range per conservation status are labeled.

## Discussion

The banded mongoose shows MHC polymorphism and selection patterns similar to those reported in other mammalian species. Remarkably, despite high levels of inbreeding, MHC diversity in this population is comparable to that of other wild carnivores not currently of conservation concern.

However, comparing MHC diversity across species requires caution. Demographic history, admixture levels, and selective pressures can all influence observed MHC diversity [24, 114, 115]. Additional factors such as sampling methods, the number of loci investigated, the number of populations included, and total sample size also play a role in shaping diversity estimates [22]. To account for these potential confounders, we applied a phylogenetic mixed model that included species identity, conservation status, sample size, MHC class, and number of exons sequenced. This analysis revealed a general trend of decreasing MHC diversity with increasing conservation threat status, suggesting that species experiencing severe population declines tend to harbor reduced immunogenetic variation, potentially compromising their adaptive potential [116–118].

Against this backdrop, the banded mongoose stands out. Despite being classified as a species of least concern, it exhibits frequent inbreeding due to strong natal philopatry, limited dispersal, and rare extra-group mating. In a separate study conducted on our long-term study population, individuals consistently showed lower microsatellite heterozygosity than expected under random mating (mean F_IT_ = 0.05, mean number of groups = 7.6, *N* = 1250; [119]). Two-thirds of individuals were inbred to some degree, and 7.1% were the result of full-sibling or parent-offspring matings [47]. Despite this, MHC diversity in banded mongooses was not lower than expected for a wild carnivore of least concern. This suggests that the forces maintaining variability at MHC loci differ from those acting on presumably neutral genomic markers. These findings are further supported by data showing a lack of significant correlation between MHC allelic diversity and genome-wide diversity (sMLH) based on 35–43 microsatellite markers [120].

Supporting this interpretation, linkage disequilibrium analysis revealed strong correlations between groups of MHC alleles, suggesting the presence of a limited number of common haplotypes in the population. Some of these haplotypes reached frequencies as high as 53% of individuals, unusually high compared to large, genetically diverse human populations, where individual haplotype frequencies are often an order of magnitude lower (Allele Frequency Net Database (AFND); [121]). In small or inbred populations, however, such patterns are not uncommon: typically polymorphic classical alleles may become nearly fixed, while nonclassical MHC genes often remain monomorphic. For instance, the isolated hunter-gatherer Maniq population in Southern Thailand shows haplotype frequencies exceeding 74%, and certain purebred dog breeds exhibit haplotypes present in more than 60% of individuals [122, 123]. These parallels suggest that the patterns observed in banded mongooses may reflect not only the presence of nonclassical loci, but also the influence of selection and their cooperative breeding system characterized by high inbreeding and relatively closed population structure.

Such dynamics are not unique to banded mongooses. A similar pattern has been observed in meerkats (*Suricata suricatta*), a closely related species with a comparable social system. In a long-term study in South Africa, 44% of meerkats had non-zero inbreeding coefficients, and 15% of individuals resulted from moderate inbreeding (F ≥ 0.125), usually in cases where mates had no prior social familiarity [124]. Despite evidence of inbreeding depression across traits such as pup mass, growth, and juvenile survival, meerkats maintained relatively high MHC-II allelic diversity ([125]; this study). Similarly, in the subterranean rodent *Ctenomys talarum*, greater MHC diversity and selection was observed in the more densely populated and inbred Mar de Cobo population compared to Necochea, where neutral genetic diversity was higher. This suggests that balancing selection may maintain MHC diversity in socially structured populations with high levels of inbreeding, even when genome-wide diversity is reduced [126]. Together, these parallels imply that cooperative breeding and social complexity may promote immunogenetic resilience in the face of inbreeding.

One non-exclusive explanation for the maintenance of MHC diversity in banded mongooses is parasite-mediated balancing selection. Group living is often associated with increased parasite transmission risk [127–129], which can intensify selection for immunogenetic diversity in social species [126, 130, 131]. Maintaining MHC diversity would enhance resistance to a broader array of pathogens, offering a fitness advantage under high parasite pressure [13–15].

Another potential mechanism is MHC-mediated sexual selection. In banded mongooses, males compete intensely during female estrus, with dominant males guarding older, more fecund females [132, 133]. Males with low MHC diversity may be in poorer condition or have higher parasite loads, reducing their competitive ability and reproductive success [134, 135]. At the same time, females exercise agency in mate selection and often breed with males other than their guards [50, 134, 135]. Mate choice for MHC-diverse partners is well documented across a range of taxa [136–138]. and may also contribute to the maintenance of MHC polymorphism in this species.

In light of the selective pressures acting on MHC genes, it is notable that the number of putative alleles and supertypes differed between MHC classes and exons in banded mongooses. MHC-I exon 2 exhibited the highest allelic and supertype diversity, followed by MHC-II DRB exon 2, while MHC-I exon 3 showed the lowest. This variation in diversity likely reflects differences in selective forces, which were also evident in the patterns of positive selection. Specifically, MHC-I exon 2 displayed the highest dN–dS ratios at putative sites under selection (PSS), followed by MHC-II DRB exon 2, and then MHC-I exon 3. These patterns are consistent with findings in other species, such as cheetahs (*Acinonyx jubatus*) and Eurasian badgers (*Meles* spp.) [22, 139], and likely result from structural and functional differences in how these exons contribute to antigen binding.

MHC-I exon 2 and exon 3 encode the α1- and α2-domains of the peptide binding region (PBR), respectively, and together form the binding groove of the MHC-I molecule [10, 140]. The α1-domain (exon 2) contains three binding pockets for peptide side chains, while the α2-domain (exon 3) contains only one [140], suggesting that selection may act more strongly on exon 2 due to its greater functional involvement in antigen presentation. These structural differences may explain the higher levels of polymorphism and positive selection at exon 2. Additionally, the higher number of noncoding sequences (pseudogenes) observed for MHC-I exon 2 may reflect ongoing birth-and-death evolution at this locus, where gene duplication produces new genes, some of which become nonfunctional due to deleterious mutations [18, 141–143].

However, direct comparisons between MHC-I and MHC-II must be interpreted cautiously. Since we sequenced the two halves of the MHC-I PBR (exon 2 and exon 3) separately, selection signals may differ across these regions, making it difficult to draw solid conclusions about class-level differences in selection. Furthermore, functional differences in pathogen binding across loci may contribute to the observed variation in selection intensity [5, 6, 144], and previous work in humans has shown stronger signatures of positive selection in MHC-I than MHC-II loci [145]. To enable more robust comparisons, future studies should sequence the complete PBR for both MHC classes, as comparing partial regions may bias interpretations of evolutionary dynamics [69].

As expected, the positively selected sites showed higher levels of dN-dS compared to the signature of selection in the full sequences for both classes and all exons. This pattern is consistent with findings in other mammals, including Eurasian beavers (*Castor fiber*) [146], and carnivores such as Namibian leopards (*Panthera pardus pardus*) [147], cheetahs (*Acinonyx jubatus*) [22], and Bengal tigers (*Panthera tigris tigris*) [40], where the peptide binding region (PBR) typically exhibits stronger signals of diversifying selection than does the whole sequence. However, in our study, using human PBR sites as a reference did not reveal stronger signals of positive selection in banded mongooses compared to the full exon sequences. This unexpected result may reflect that human PBR sites are not fully conserved or functionally equivalent in mongooses. Nonetheless, this explanation is not entirely satisfactory, as previous research has demonstrated substantial structural conservation of the PBR between humans and felids [148, 149].

To further investigate MHC evolution in banded mongooses, we constructed gene sequence trees including related carnivore species. These phylogenies showed clear suborder-level clustering, with feliforms (e.g., mongooses, cats) separating from caniforms. suggesting more recent, species-specific duplication events. In contrast, MHC-II DRB and DQB loci grouped across broader taxonomic levels, indicating a deeper evolutionary history. This distinction between MHC-I and II evolutionary patterns is consistent with previous work highlighting the longer evolutionary trajectory of MHC-II loci [150].

Phylogenetic analysis also revealed that some nonclassical loci are shared among felids, including cats, mongooses, and meerkats, suggesting the presence of trans-species polymorphism. At the same time, some nonclassical loci, such as FLAI-J and FLAI-L, appear species-specific. Molecular comparisons between conserved HLA sites and sequences recovered in this study support the presence of both nonclassical MHC-I and MHC-II loci in the banded mongoose. This distinction is important for downstream analyses, as nonclassical loci are typically not involved in antigen presentation [7–9] and may confound associations between MHC diversity and fitness.

Phylogenetic tree inference, the number of putative alleles found per individual, and linkage disequilibrium analysis indicate that banded mongooses have at least 6 MHC-I loci and 4 MHC-II loci. However, we were not able to infer the number of loci from our data with the current methods despite relying on a thorough validation pipeline, a well-established NGS protocol with technical replicates of every sample, negative controls on each plate, and plausibility checks with pedigree data and residues with structural importance. This could be caused by large differences in the number of sequences recovered per individual. Causes for such large variation could be either copy-number variation (like in cats *Felis catus* [95]) or amplification bias during PCR. Assignment of the putative alleles to loci could help reveal patterns of copy-number variation as well as enable estimating heterozygosity levels, determining whether alleles are shared across loci, and investigating potential inter-locus gene conversion. Amplification bias was anticipated when designing this study, and we tried to avoid uneven distribution of reads by using a normalization kit. However, this only prevents amplification bias between samples but not between loci. Locus-specific primers developed from fully resolved banded mongoose genomes would answer this question, and this is a goal for the near-future.

## Conclusion

Despite high inbreeding, banded mongooses maintain MHC diversity comparable to other carnivores of least conservation concern, challenging the view that inbreeding inevitably reduces immunogenetic variation. This resilience may be driven by parasite-mediated balancing selection, MHC-based mate choice, or other mechanisms operating in socially structured populations. High-throughput sequencing revealed marked differences between the two halves of the MHC-I binding groove, with exon 2 showing greater diversity and stronger diversifying selection than exon 3. Phylogenetic analyses uncovered nonclassical loci in both MHC classes, including sequences clustering with classical loci, suggesting species-specific gene duplication, as well as trans-species polymorphism indicating shared ancestry or convergent evolution. Together, these findings provide one of the most comprehensive assessments of MHC diversity in a wild carnivore, highlighting complex evolutionary dynamics and the need to link historical selection to contemporary processes such as mate choice, reproduction, and survival.

## Supplementary Information

Supplementary Material 1.

## Data Availability

Data and R code used in this paper can be found at the Figshare Repository: 10.6084/m9.figshare.29827823.v1. Sequences analyzed during the current study have been deposited to NCBI GenBank (http://www.ncbi.nlm.nih.gov/genbank) under the GenBank accession #s: PQ137681 - PQ137748.
