# Supplementary material for: Inbreeding does not reduce major histocompatibility complex diversity in the banded mongoose
Source: BMC Ecol Evol. 2025 Oct 14;25:104. doi: 10.1186/s12862-025-02456-x (PMC12519839; doi:10.1186/s12862-025-02456-x)
Supplement: Supplementary file 1 — Supplementary Material 1. [file 12862_2025_2456_MOESM1_ESM.pdf]

BMC Ecology and Evolution

Inbreeding does not reduce major histocompatibility complex diversity in the banded mongoose

Nadine Schubert, Hazel J. Nichols, Francis Mwanguhya, Robert Businge, Solomon Kyambulima, Kenneth Mwesige, Michael A. Cant, Jamie C. Winternitz✉

✉ Jamie C. Winternitz
Department of Evolutionary Immunogenomics
Institute for Animal Cell and Systems Biology
University of Hamburg, Germany
Email: jcwinternitz@gmail.com

SUPPLEMENTARY MATERIAL AND METHODS

Table of Contents

METHODS 2
PCR amplification and NGS library construction 2
Assigning alleles to loci 2
Table S1. Carnivore sequences used for development of MHC primers 3
Table S2. Genotyping primers 6
Table S3. AmpliSAS settings 6
Table S4. Conserved site comparison 7
Table S5. CODEML model comparison per exon 8
Table S6. MHC-I comparison of human PBR, FUBAR + MEME and CODEML PSS 9
Table S7. MHC-II comparison of human PBR, FUBAR + MEME and CODEML PSS 10
Figure S1. Flow chart for artifact sequence identification 11
Figure S2. Supertype frequencies for MHC-I and II 12
Figure S3. MHC allele accumulation curves 13
Figure S4. Consensus species tree 14
REFERENCES 15

METHODS

PCR amplification and NGS library construction

PCRs were carried out with a total reaction volume of 20 µl containing 14.3 µl of DNase and RNase free water, 2 µl of Buffer S (VWR, United States), 0.5 µl 40 mM dNTPs (Carl Roth, Germany), 0.5 µl of 10 µM of the tagged forward and reverse primers, 0.2 µl of Taq polymerase, and 2 µl of DNA (corresponding to ~40-100 ng of DNA). The PCRs were run on cyclers according to the following protocol: denaturation at 94°C for 2 min, followed by 30 cycles of 94°C for 30 seconds, annealing at either 55°C for MHC-I exon 2 and 3 or 58°C for MHC-II DRB exon 2 for 30 seconds, and elongation at 72°C for 60 seconds, and finally 10 min of elongation at 72°C and cooling down to a storage temperature of 4°C. We monitored the success of the PCR by running 5 µl of the PCR product on a 2% agarose gel.

We used the NGS Normalization 96-well kit (Norgen Biotek Corp., Canada) according to the manufacturers protocol to clean up the PCR products and simultaneously adjust amplicon concentrations. We then pooled the normalized samples per plate for preparation of libraries and measured the amplicon concentrations of the pools using the Qubit® dsDNA High Sensitivity Assay Kit on a Qubit® 2.0 Fluorometer (Invitrogen/Thermo Fisher, United States). We used the Illumina TruSeq DNA Nano Low Throughput Library Prep Kit (Illumina Inc, United States) in combination with the TruSeq DNA Single Indexes Set A and B (Illumina Inc, United States). Some indexes were used for multiple libraries, but together with the unique combination of tags and primer sequences, we ensured identification of samples during high-throughput sequencing. We followed the manufacturers protocol for library preparation with some minor adjustments. Due to lower concentrations of the pooled amplicon samples (ranging from 0.04 ng/µl to 0.3 ng/µl) we increased the amplicon volume in the end repair step to 200 µl instead of 50 µl. Furthermore, we prepared dilutions of the indexes used for each pool according to the amplicon concentration with an undiluted index volume being adjusted to 100 ng of DNA to avoid dimers.

We checked library quality using the Agilent Bioanalyzer 2100 (Agilent Technologies, United States) together with the High Sensitivity DNA Kit (Agilent Technologies, United States). Despite the adjustment of the index concentration, we detected a peak comprised of index dimers for some libraries and thus performed gel extraction on these libraries to separate the amplicon from the index dimers. Therefore, we ran the libraries for 3h at 65V on a 1.5% agarose gel, cut out the bands with the correct amplicon size and then used either the innuPREP Gel Extraction Kit (Analytik Jena, Germany) or the QIAquick Gel Extraction Kit (Qiagen, the Netherlands) according to the manufacturer's protocol. A final quality control using the Agilent Bioanalyzer 2100 with the High Sensitivity DNA Kit was carried out before preparation of the libraries for high-throughput sequencing. Afterwards, all libraries were pooled into one tube equimolarly with initial amplicon concentrations ranging from 1.1 pmol/l to 9.4 pmol/l.

Assigning alleles to loci

We used MHC typer V1.1, which uses a maximum-likelihood approach for reconstructing haplotypes while considering null alleles or copy number variation (CNV), identical alleles shared between loci as well as deviations from Hardy-Weinberg-Equilibrium [1]. The protocol used was the recommended 2-step procedure [1]: step 1: random initial solution for #loci=8 for MHC-I exon 2, 3 for MHC-I exon 3 and 4 for MHC-II DRB exon 2, number of repeats=10, chain length=500, initial temperature=0.01, final temperature=0.00001, anneal coefficient=0.99, max iteration=30, min freq. diff=0.002, penalty of missing=-2, penalty of mismatch=-80, taboo coef.=1; Step 2: initial solution for loci=solution from best run of Step 1,

number of repeats=300, initial temperature=0.0001, final temperature=0.000001, consider null alleles, consider deviation from HWE, initial null allele freq.=0.05, penalty of missing=0, penalty of mismatch=-1000, taboo coef.=1.00001. We considered an assignment to loci as reliable when a run resulted in the same assignment at least twice with an optimal Bayesian information criterion (BIC).

Table S1. Carnivore sequences used for development of MHC primers

Class I exon 2 Primer Pair: MHCI-alpha1F, 1787R & MHCI-alpha1F, 1793R

Accession	Common Name	Description	Organism	Length bp
GU971407	cheetah	Acinonyx jubatus MHC class I antigen (Acju-MHCI) gene, Acju-MHCI*02 allele, exons 2, 3 and partial cds	Acinonyx jubatus	714
EU162656	giant panda	Ailuropoda melanoleuca MHC class I antigen (Aime-128) gene, complete cds	Ailuropoda melanoleuca	4036
LC189199	dog	Canis lupus familiaris DLA-88b gene for MHC class I antigen, complete cds, allele: DLA-88*017:01	Canis lupus familiaris	5538
KC763064	domestic cat	Felis catus MHC class I antigen (FLAI-E) gene, FLAI-E-FLA-E*00501 allele, exons 1 through 4 and partial cds	Felis catus	1908
JX218916	gray seal	Halichoerus grypus MHC class I antigen (HagrN) gene, HagrN*05:05 allele, partial cds	Halichoerus grypus	2777
AY909914	Asiatic lion	Panthera leo persica clone FM622 MHC class I antigen gene, exon 2 and partial cds	Panthera leo persica	266
JX218894	harbor seal	Phoca vitulina MHC class I antigen (PhviN) gene, PhviN*06:01:01 allele, partial cds	Phoca vitulina	3266

Class I exon 3 Primer Pair: 2000F, 2269R

Accession	Common Name	Description	Organism	Length bp
AF283086	Pallas's cat	Otocolobus manul MHC class I antigen (FLA-Z) gene, FLA-Z*1 allele, exon 3 and partial cds	Otocolobus manul	218
AF283087	sand cat	Felis margarita MHC class I antigen (FLA-Z) gene, FLA-Z*1 allele, exon 3 and partial cds	Felis margarita	218
AF283090	Asian golden cat	Catopuma temminckii MHC class I antigen (FLA-Z) gene, FLA-Z*1 allele, exon 3 and partial cds	Catopuma temminckii	218
AF283091	margay	Leopardus wiedii MHC class I antigen (FLA-Z) gene, FLA-Z*2 allele, exon 3 and partial cds	Leopardus wiedii	218
AF283095	caracal	Caracal caracal MHC class I antigen (FLA-Z) gene, FLA-Z*5 allele, exon 3 and partial cds	Caracal caracal	218
AF283099	ocelot	Leopardus pardalis MHC class I antigen (FLA-Z) gene, FLA-Z*10 allele, exon 3 and partial cds	Leopardus pardalis	218
AF283100	Geoffroy's cat	Oncifelis geoffroyi MHC class I antigen (FLA-Z) gene, FLA-Z*8 allele, exon 3 and partial cds	Leopardus geoffroyi	218
AF283101	domestic cat	Felis catus MHC class I antigen (FLA-Z) gene, FLA-Z*9 allele, exon 3 and partial cds	Felis catus	218
AY910008	Asiatic lion	Panthera leo persica clone S1023 MHC class I antigen gene, exon 3 and partial cds	Panthera leo persica	246
EU162656	giant panda	Ailuropoda melanoleuca MHC class I antigen (Aime-128) gene, complete cds	Ailuropoda melanoleuca	4036
GU971407	cheetah	Acinonyx jubatus MHC class I antigen (Acju-MHCI) gene, Acju-MHCI*02 allele, exons 2, 3 and partial cds	Acinonyx jubatus	714

JX218894	harbor seal	Phoca vitulina MHC class I antigen (PhviN) gene, PhviN*06:01:01 allele, partial cds	Phoca vitulina	3266
JX218916	gray seal	Halichoerus grypus MHC class I antigen (HagrN) gene, HagrN*05:05 allele, partial cds	Halichoerus grypus	2777
KC763064	domestic cat	Felis catus MHC class I antigen (FLAI-E) gene, FLAI-E-FLA-E*00501 allele, exons 1 through 4 and partial cds	Felis catus	1908
LC189199	dog	Canis lupus familiaris DLA-88b gene for MHC class I antigen, complete cds, allele: DLA-88*017:01	Canis lupus familiaris	5538

Class II exon 2 Primer Pair: 142F, 429R & 188F, 450R

**Accession**	**Common Name**	**Description**	**Organism**	**Length bp**
AB490457	brown bear	Ursus arctos MHC-DRBgene, exon 2, partial sequence, allele: Urar-DRB*01	Ursus arctos	267
AB490478	Asiatic black bear	Ursus thibetanus MHC-DRBgene, exon 2, partial sequence, allele: Urth-DRB*01	Ursus thibetanus	267
AB490480	American black bear	Ursus americanus MHC-DRBgene, exon 2, partial sequence, allele: Uram-DRB*01	Ursus americanus	249
AB490483	Malayan sun bear	Helarctos malayanus MHC-DRBgene, exon 2, partial sequence, allele: Urml-DRB*01	Helarctos malayanus	249
AB490485	sloth bear	Melursus ursinus MHC-DRBgene, exon 2, partial sequence, allele: Urur-DRB*01	Melursus ursinus	249
AB490489	spectacled bear	Tremarctos ornatus MHC-DRBgene, exon 2, partial sequence, allele: Tror-DRB*01	Tremarctos ornatus	249
AF016910	dog	Canis familiaris MHC class II antigen (DRB) gene, DRB1*010011 allele, exon 2 and partial cds	Canis lupus familiaris	237
AF057808	ocelot	Leopardus pardalis MHC class II DR antigen beta subunit (Lepa-DRB) gene, Lepa-DRB*0201 allele, exon 2 and partial cds	Leopardus pardalis	238
AF057913	margay	Leopardus wiedii MHC class II DR antigen beta subunit (Lewi-DRB) gene, Lewi-DRB*0201 allele, exon 2 and partial cds	Leopardus wiedii	235
AF458914	polar bear	Ursus maritimus MHC class II DR alpha 1 domain (Urma-DRB) gene, Urma-DRB*01 allele, exon 2 and partial cds	Ursus maritimus	237
AF516916	red wolf	Canis rufus MHC class II histocompatibility antigen (Caru-DRB) gene, Caru-DRB*1 allele, partial cds	Canis rufus	280
AF516920	coyote	Canis latrans MHC class II histocompatibility antigen (Cala-DRB) gene, Cala-DRB*13 allele, partial cds	Canis latrans	280
AJ428209	domestic cat	Felis catus partial FLA-DRB gene for MHC class II antigen, DRB1-rr2 allele, exon 2	Felis catus	237
AY312960	cheetah	Acinonyx jubatus MHC class II antigen (FLA-DRB1) gene, FLA-DRB1*ha14 allele, exon 2 and partial cds	Acinonyx jubatus	238
AY491457	California sea lion	Zalophus californianus MHC class II antigen (Zaca-DRB) mRNA, Zaca-DRB*02 allele, partial cds	Zalophus californianus	863
M29611	dog	C.familiaris MHC class II DLA-DR-beta-1 gene, complete cds	Canis lupus familiaris	1165
DQ189257	Clouded leopard	Neofelis nebulosa MHC class II antigen (Nene-DRB) gene, Nene-DRB*1 allele, exon 2 and partial cds	Neofelis nebulosa	237
DQ189261	tiger	Panthera tigris MHC class II antigen (Pati-DRB) gene, Pati-DRB*1 allele, exon 2 and partial cds	Panthera tigris	237
DQ189262	leopard	Panthera pardus MHC class II antigen (Papa-DRB) gene, Papa-DRB*1 allele, exon 2 and partial cds	Panthera pardus	237

DQ354689	Hooker's sea lion	Phocarctos hookeri MHC class II antigen (Phho93-32-DRB) gene, Phho93-32-DRB*0402 allele, exon 2 and partial cds	Phocarctos hookeri	216
EF125965	giant panda	Ailuropoda melanoleuca MHC class II antigen DRB gene, partial cds	Ailuropoda melanoleuca	281
EF690694	Arctic fox	Vulpes lagopus MHC class II antigen (Vula-DRB) gene, Vula-DRB*1 allele, partial cds	Vulpes lagopus	246
EU916192	domestic cat	Felis catus clone DRB*n01 MHC class II antigen beta chain (Feca-DRB) mRNA, complete cds	Felis catus	801
EU918395	Eurasian lynx	Lynx lynx MHC class II antigen (Lyly-DRB) gene, Lyly-DRB*01 allele, exon 2 and partial cds	Lynx lynx	238
FJ210684	snow leopard	Uncia uncia MHC class II antigen (Paun-DRB) gene, Paun-DRB*201 allele, exon 2 and partial cds	Panthera uncia	237
FJ210685	leopard cat	Prionailurus bengalensis MHC class II antigen (Prbe-DRB) gene, Prbe-DRB*201 allele, exon 2 and partial cds	Prionailurus bengalensis	237
FJ210694	Asiatic golden cat	Catopuma temminckii MHC class II antigen (Pate-DRB) gene, Pate-DRB*201 allele, exon 2 and partial cds	Catopuma temminckii	237
FJ210695	Pallas's cat	Otocolobus manul MHC class II antigen (Otma-DRB) gene, Otma-DRB*201 allele, exon 2 and partial cds	Otocolobus manul	237
FJ210697	lion	Panthera leo MHC class II antigen (Pale-DRB) gene, Pale-DRB*201 allele, exon 2 and partial cds	Panthera leo	237
FJ210702	Chinese desert cat	Felis silvestris bieti MHC class II antigen (Febi-DRB) gene, Febi-DRB*101 allele, exon 2 and partial cds	Felis silvestris bieti	237
GU388312	raccoon	Procyon lotor MHC class II antigen (Prlo-DRB) gene, Prlo-DRB*01 allele, exon 2 and partial cds	Procyon lotor	228
HM004073	fish-eating bat	Myotis vivesi MHC class II antigen (Myvi-DRB) mRNA, Myvi-DRB-Myvi12 allele, partial cds	Myotis vivesi	330
KP127615	Australian sea lion	Neophoca cinerea MHC class II antigen (NeciDRB) gene, NeciDRB*01 allele, partial cds	Neophoca cinerea	226
KU059084	American badger	Taxidea taxus MHC class II antigen (DRB) gene, DRB-H01 allele, exon 2 and partial cds	Taxidea taxus	227
LC055006	Siberian weasel	Mustela sibirica Musi-DRB gene for MHC class II antigen, Musi-DRB*01_ allele, exon 2 and partial cds	Mustela sibirica	242
LC055117	Japanese weasel	Mustela itatsi Muit-DRB gene for MHC class II antigen, Muit-DRB*01_ allele, exon 2 and partial cds	Mustela itatsi	242
LC174990	least weasel	Mustela nivalis Muni-DRB gene for MHC class II antigen, partial cds, allele: Muni-DRB*01	Mustela nivalis	242
LN832041	Eurasian red squirrel	Sciurus vulgaris partial mRNA for MHC class II DRB (Scvu-DRB2 gene), allele Scvu-DRB2a	Sciurus vulgaris	367
U51543	little spotted cat	Leopardus tigrinus MHC class II DRB first extracellular domain MHCLETI-DRB gene (DRB*0501 allele), partial cds	Leopardus tigrinus	238
U51548	Geoffroy's cat	Oncifelis geoffroyi MHC class II DRB first extracellular domain MHCONGE-DRB gene (DRB*0201 allele), partial cds	Leopardus geoffroyi	238
U51546	Iriomote cat	Prionailurus bengalensis iriomotensis MHC class II DRB first extracellular domain MHCMAIR-DRB gene (DRB*0401 allele), partial cds	Prionailurus iriomotensis	238
HQ230582	spotted hyena	Crocuta crocuta voucher kc 29 clone 37 MHC class II antigen (MHC-DRB) gene, exon 2 and partial cds	Crocuta crocuta	198

HQ230583	spotted hyena	Crocuta crocuta voucher kc 29 clone 38 MHC class II antigen (MHC-DRB) gene, exon 2 and partial cds	Crocuta crocuta	198
JN985763	striped hyena	Hyaena hyaena clone 2_4 MHC class II antigen (Hyhy-DRB) gene, exon 2 and partial cds	Hyaena hyaena	201
JN985764	striped hyena	Hyaena hyaena clone 4_13 MHC class II antigen (Hyhy-DRB) gene, exon 2 and partial cds	Hyaena hyaena	201

Table S2. Genotyping primers

MHC Class and exon	Label	Type	Sequence 5'-->3'	Tm	Product size (w/o primers)	Reference
MHC-I exon 2	MHCI-alpha1F	F	CCACTCCCTGAGGTATTTCTACACC	60.7-62.1	221	
	1787R	R	GGCCTCGCTCTGGTTGTAG	60.2		[2]
MHC-I exon 2	MHCI-alpha1F	F	CCACTCCCTGAGGTATTTCTACACC	60.7-62.1	228	
	1793R	R	CTCACCGGCCTCGCTCTG	62.5		[2]
MHC-I exon 3	2000F	F	GGTCACACAGCATCCAGAGA	59.4	232	
	2269R	R	GCTGCAGCGTCTCCTTCC	60.8		Newly made
MHC-II DRB exon 2	188F	F	CGAGTGCCATTTCACCAACG	60.1	201	
	429R	R	GCTGCACCGTGAAGCTCT	60.4		Newly made
MHC-II DRB exon 2	JS1	F	GAGTGTCATTTCTACAACGGGAC	59.3	171	
	JS2	R	GATCCCGTAGTTGTGTYTGCA	58.9-60.1		[3]

Selected primer sets are shaded blue. The PCR profiles consisted of the following: (MHC-I) 94°C for 2 minutes, then 30 cycles of denaturing at 94°C for 30 seconds, annealing at 55°C for 30 seconds, extension at 72°C for 30 seconds, and at the end holding at 72°C for 10 minutes; (MHC-II) 94°C for 2 minutes, then 30 cycles of denaturing at 94°C for 30 seconds, annealing at 58°C for 30 seconds, extension at 72°C for 30 seconds, and at the end holding at 72°C for 10 minutes.

Table S3. AmpliSAS settings

Parameter	MHCI exon 2	MHCI exon 3	MHCII DRB exon 2
substitution_threshold	1	1	1
indel_threshold	0.001	0.001	0.001
cluster_exact_length	1	1	1
cluster_inframe	1	1	1
min_dominant_frequency_threshold	25	25	25
min_amplicon_depth	100	100	100
min_amplicon_seq_frequency	1.4	1.6	1.2
discard_frameshifts	1	1	1
min_chimera_length	10	10	10
max_allele_number	16	10	10

Table S4. Conserved site comparison

MHC class and exon	[4]	Location in HLA-A2 (AAA76608.2) starting from exon 1	Mumu allele status
MHC-I exon 2	Y7	Y31	Not covered by amplicon
	Y59	Y83	Mumu-class-I-2*05 and *24 with F, *26 and *37 with H
	Y84	Y108	all conserved
MHC-I exon 3	T143	T167	all conserved
	K146	K170	all conserved
	W147	W171	all conserved
	Y159	Y183	all conserved
	Y171	Y195	Mumu-class-I-3*07, *09, *13 and *14 with H
		**Location in HLA-DRB1 (NP_002115.2)**	
MHC-II DRB exon 2	W61	W90	Mumu-DRB*07 and *11 with R
	H81	H110	all conserved
	N82	N111	all conserved

Table S5. CODEML model comparison per exon

MHC class and exon	Site Model	np	Ln L	Estimates of parameters			Models compared	LRT P-value	Positive sites
MHC-I exon 2	M8	58	-1455.420531	p0=0.76768<br>(p1= 0.23232)	p=0.72301<br>ω= 2.00786	q=2.07887	M7 vs.M8	0.000170334	14E 0.990*, 32S 0.521, 35V 0.695, 37P 0.579, 56R 0.807, 57V 0.995**, 60T 0.963*, 63M 0.990**, 67S 0.901, 69Q 0.997**, 71A 0.995**, 73G 0.784
	M7	56	-1464.098280	p=0.06839		q=0.08242			Not Allowed
	M8a	57	-1461.112431	p0=0.69365<br>(p1= 0.30635)	p=1.32201<br>ω= 1.00000	q=8.62292	M8a vs.M8	0.000740874	Not Allowed
MHC-I exon 3	M8	22	-508.578835	p0=0.96110<br>(p1= 0.03890)	p=0.04292<br>ω= 30.62627	q=0.05700	M7 vs.M8	0.000114224	17S 0.987*, 19L 0.882, 58R 0.995**, 59Y 0.999**, 66S 0.719
	M7	20	-517.656182	p=0.03707		q=0.05070			Not Allowed
	M8a	21	-517.282231	p0=0.76818<br>(p1= 0.23182)	p=4.49321<br>ω= 1.00000	q=99.00000	M8a vs.M8	0.000030175	Not Allowed
MHC-II DRB exon 2	M8	28	-1024.201775	p0=0.74954<br>(p1= 0.25046)	p=0.25697<br>ω= 2.02009	q=1.33606	M7 vs.M8	0.017350586	6F 0.695, 8D 0.741, 10Y 0.997**, 17Y 0.944, 18V 0.502 , 37S 0.652, 44L 0.957*, 47R 1.000**, 50D 0.969*, 51A 0.686, 54A 0.763, 57W 0.771, 58Y 0.833, 66A 0.946
	M7	26	-1028.255904	p=0.06002		q=0.07705			Not Allowed
	M8a	27	-1028.306510	p0=0.68254<br>(p1= 0.31746)	p=0.35303<br>ω= 1.00000	q=4.43357	M8a vs.M8	0.004167232	Not Allowed

Table S6. MHC-I comparison of human PBR, FUBAR + MEME and CODEML PSS

Exon	Saper et al. 1991 HLA-A2 from Table 14. [5]	Position including signal peptide (exon 1)	Position in banded mongoose sequence	PSS overlap	FUBAR + MEME PSS	CODEML significant PSS[1]
Exon 2 (α1 = 1-90 aa)	5	29			4	__14 E 0.990**__
	7	31			14	__57 V 0.995**__
	9	33	-2		35	60 T 0.963*
	24	48	14	yes	37	63 M 0.990**
	25	49	15		57	69 Q 0.997**
					64	71 A 0.995**
	34	58	24		67	
	45	69	35	yes	72	
	59	83	49			
	63	87	53			
	66	90	56			
	67	91	57	yes		
	70	94	60			
	73	97	63			
	74	98	64	yes		
	77	101	67	yes		
	80	104	70			
	81	105	71			
	84	108	74			
Exon 3 (α2 = 91-182 aa)	97	121	-1		59	17 S 0.987*
	99	123	2		66	58 R 0.995**
	114	138	17			__59 Y 0.999**__
	116	140	19			
	123	147	26			
	143	167	46			
	146	170	49			
	147	171	50			
	150	174	53			
	152	176	55			
	155	179	58			
	156	180	59	yes		
	159	183	62			
	160	184	63			
	163	187	66	yes		
	167	191	70			
	171	195	74			

[1]CODELML sites in bold match banded mongoose PSS; underlined sites match human PBR

Table S7. MHC-II comparison of human PBR, FUBAR + MEME and CODEML PSS

Brown et al. 1993 DRB1*0101 from caption of Fig. 3. [6]	Position including signal peptide (exon 1)	Position in banded mongoose sequence	PSS overlap	FUBAR + MEME PSS	CODEML significant PSS[1]
9	38			8	10 Y 0.997**
11	40			18	**44 L 0.957***
13	42			44	47 R 1.000**
28	57	8	yes	50	**50 D 0.969***
30	59	10		57	66 A 0.946*
32	61	12			
37	66	17			
38	67	18	yes		
47	76	27			
56	85	36			
60	89	40			
61	90	41			
65	94	45			
68	97	48			
70	99	50	yes		
71	100	51			
74	103	54			
78	107	58			
81	110	61			
82	111	62			
85	114	65			
86	115	66			
88	117	68			
89	118	69			

[1]CODELML sites in bold match banded mongoose PSS; underlined sites match human PBR

Flow chart – artifact identification

blue = keep sequence, orange = remove sequence

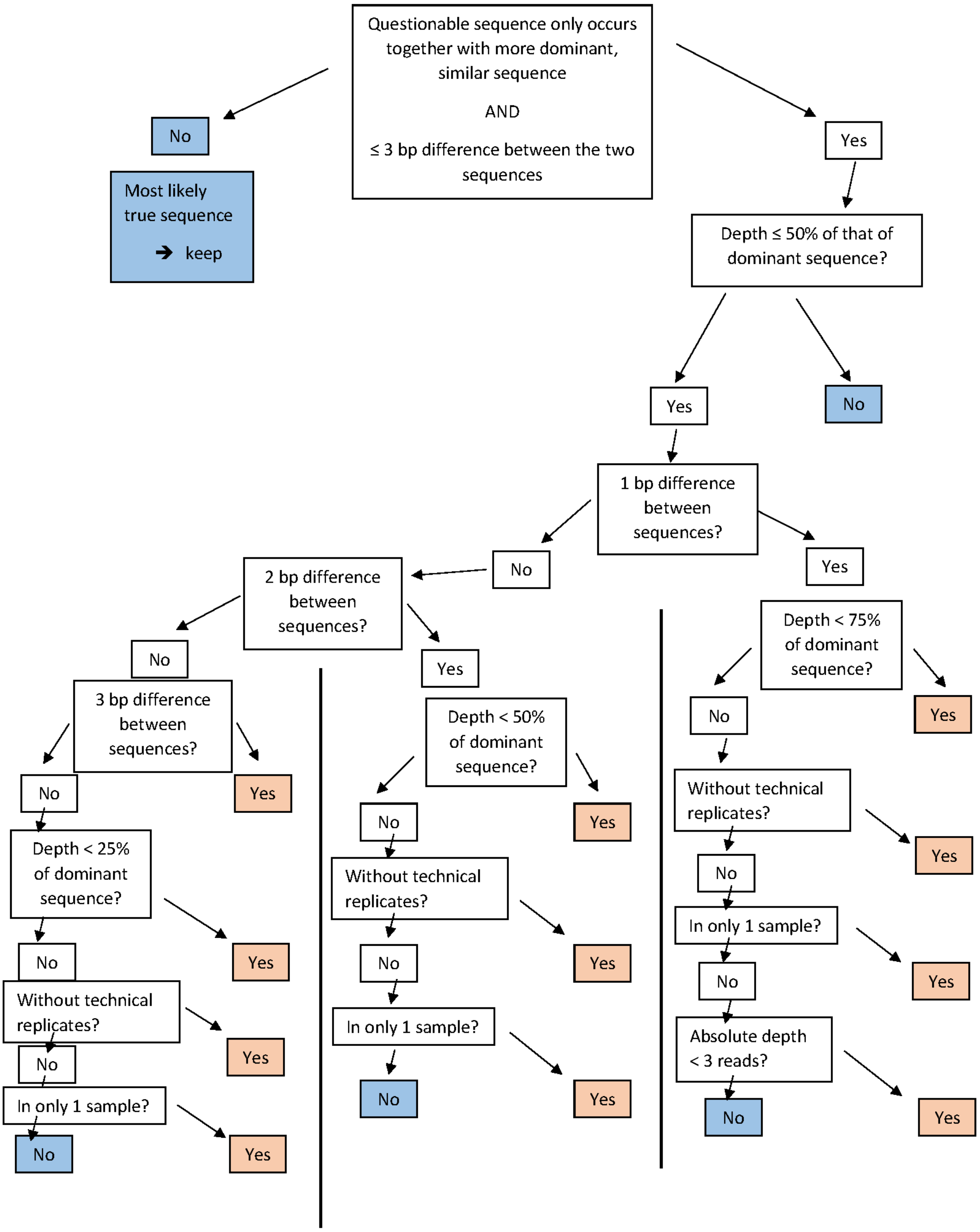

Figure S1. Flow chart for artifact sequence identification

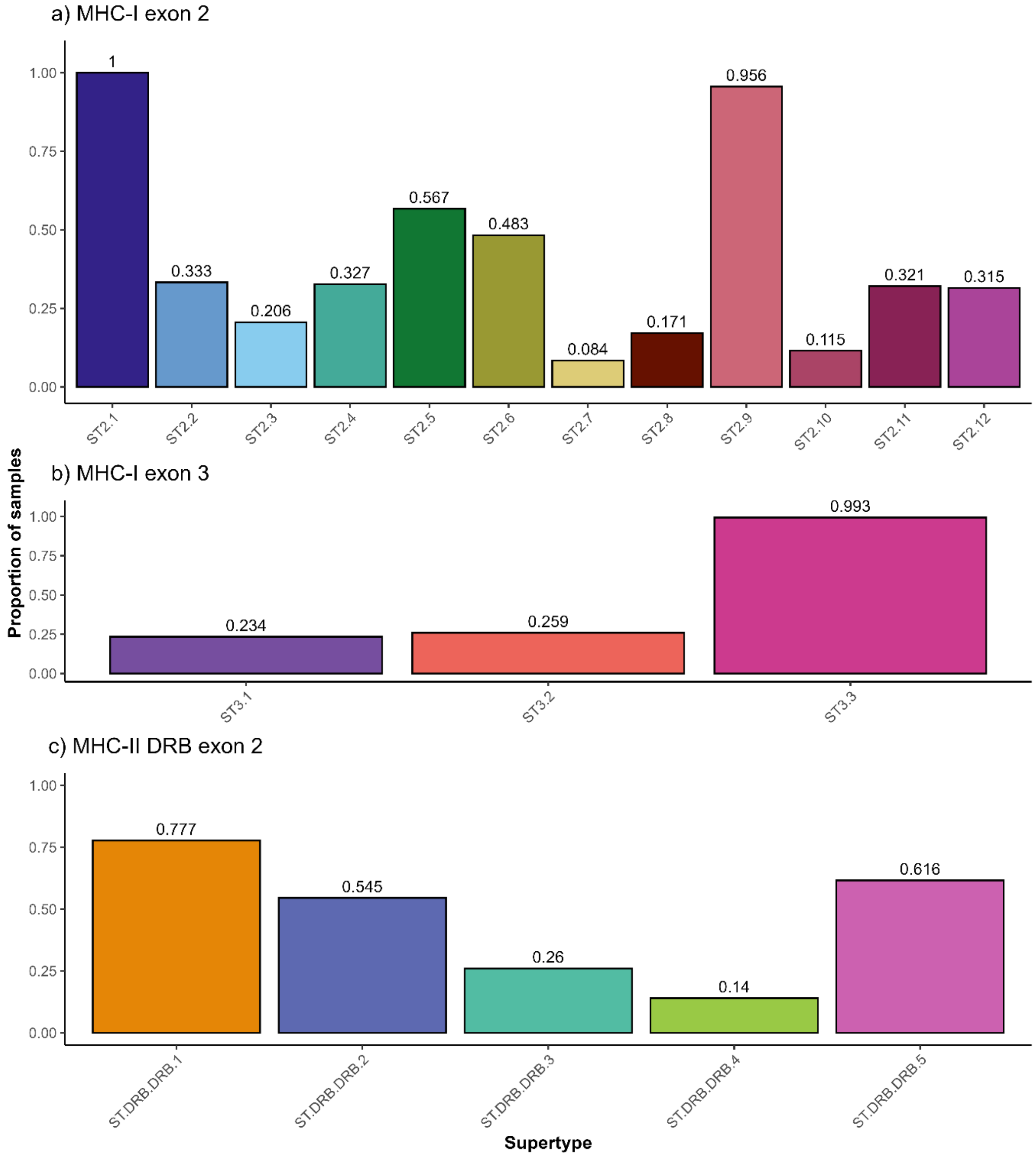

Figure S2. Supertype frequencies for MHC-I and II

The proportion of individuals with each supertype is shown. Proportions range from 0.084 (27/321 individuals) to 1 (321 individuals) for MHC-I exon 2 (a), 0.234 (66/282 individuals) to 0.993 (280/282 individuals) for MHC-I exon 3 (b) and 0.14 (54/385 individuals) to 0.777 (299/385 individuals) for MHC-II DRB exon 2 (c).

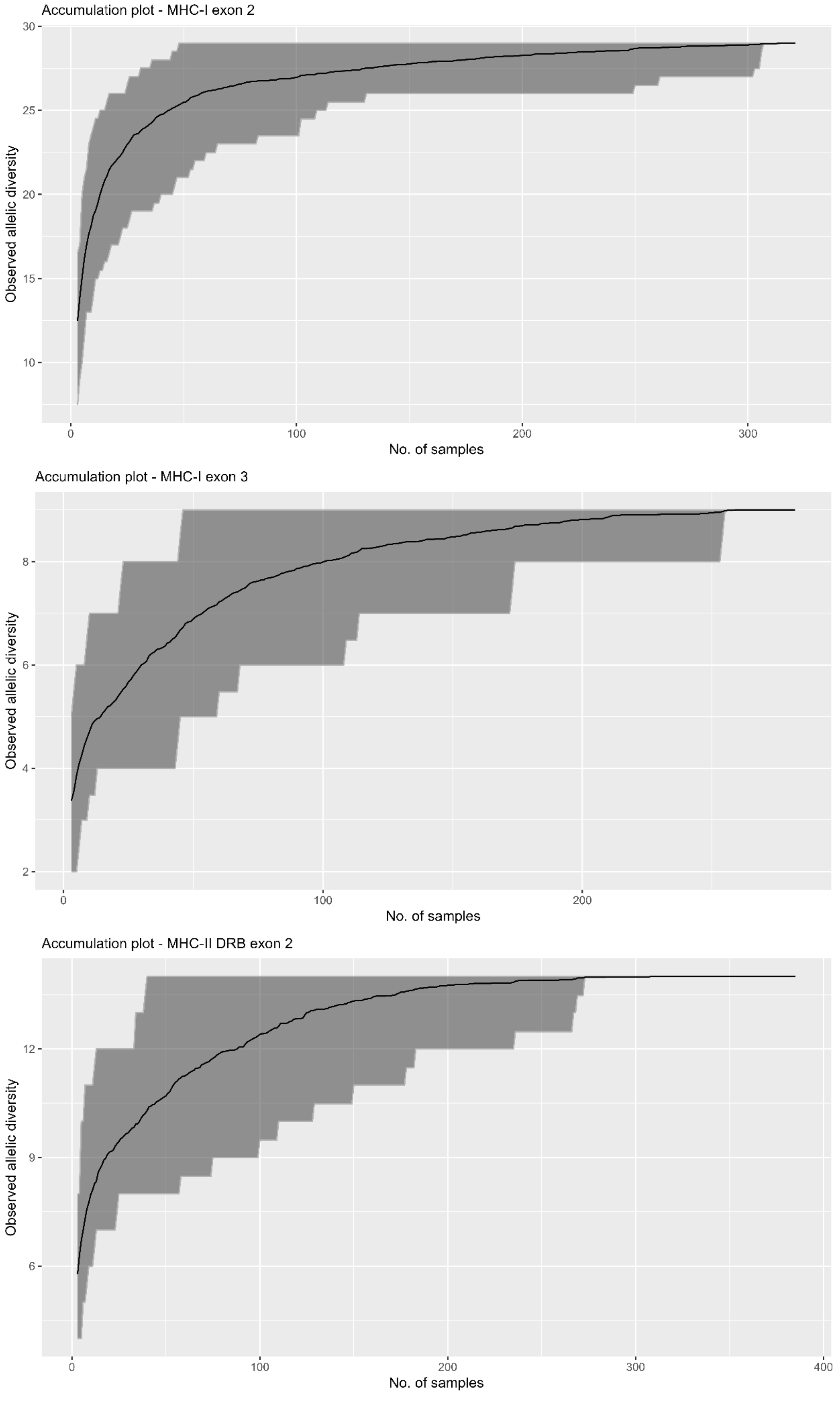

Figure S3. MHC allele accumulation curves

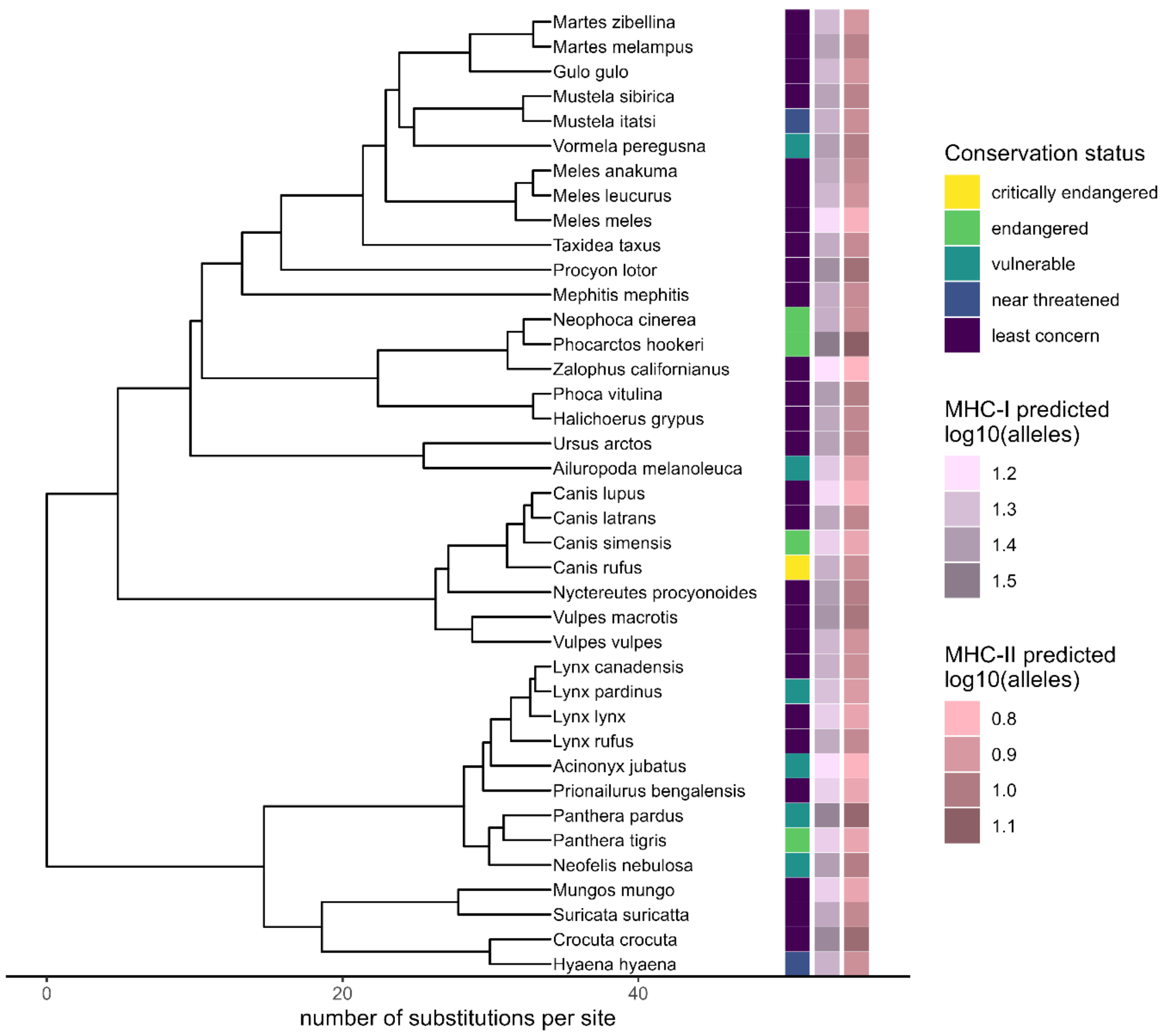

Figure S4. Consensus species tree

Consensus phylogenetic tree used for Bayesian phylogenetic linear mixed model with conservation status and allelic diversity per MHC class, controlling for conservation status, phylogeny, MHC class, and sampling effort (number of individuals and exons).

REFERENCES

1. Huang K, Zhang P, Dunn DW, Wang T, Mi R, Li B: Assigning alleles to different loci in amplifications of duplicated loci. *Molecular Ecology Resources* 2019, 19:1240–1253.
2. Yuhki N, O'Brien SJ: DNA recombination and natural selection pressure sustain genetic sequence diversity of the feline MHC class I genes. *Journal of Experimental Medicine* 1990, 172(2):621-630.
3. Schad J, Sommer S, Ganzhorn J: MHC variability of a small lemur in the littoral forest fragments of southeastern Madagascar. *Conservation Genetics* 2004, 5(3):299-309.
4. Kaufman J, Salomonsen J, Flajnik M: Evolutionary conservation of MHC class I and class II molecules - different yet the same. *Seminars in Immunology* 1994, 6(6):411-424.
5. Saper MA, Bjorkman PJ, Wiley DC: Refined structure of the human histocompatibility antigen HLA-A2 at 2.6 Å resolution. *Journal of Molecular Biology* 1991, https://doi.org/10.1016/0022-2836(91)90567-P.
6. Brown JH, Jardetzky TS, Gorga JC, Stern LJ, Urban RG, Strominger JL, Wiley DC: Three-dimensional structure of the human class II histocompatibility antigen HLA-DR1. *Nature* 1993, https://doi.org/10.1038/364033a0.
